# An Orally Bioavailable and Highly Efficacious Inhibitor of CDK9/FLT3 for the Treatment of Acute Myeloid Leukemia

**DOI:** 10.3390/cancers14051113

**Published:** 2022-02-22

**Authors:** Abel Tesfaye Anshabo, Laychiluh Bantie, Sarah Diab, Jimma Lenjisa, Alemwork Kebede, Yi Long, Gary Heinemann, Jasmine Karanjia, Benjamin Noll, Sunita K. C. Basnet, Manjun Li, Robert Milne, Hugo Albrecht, Shudong Wang

**Affiliations:** Drug Discovery and Development, Clinical and Health Sciences, University of South Australia, Adelaide, SA 5001, Australia; abel_tesfaye.anshabo@mymail.unisa.edu.au (A.T.A.); laychiluh.mekonnen@unisa.edu.au (L.B.); diab.s@wehi.edu.au (S.D.); jimma.lenjisa@unisa.edu.au (J.L.); alemwork.kebede@unisa.edu.au (A.K.); yi.long@unisa.edu.au (Y.L.); gary.heinemann@unisa.edu.au (G.H.); jasmine.karanjia@mymail.unisa.edu.au (J.K.); ben.noll@unisa.edu.au (B.N.); sunita.kcbasnet@unisa.edu.au (S.K.C.B.); manjun.li@unisa.edu.au (M.L.); robert.milne@unisa.edu.au (R.M.)

**Keywords:** CDK9, FLT3, targeted therapy, cancer, leukemia

## Abstract

**Simple Summary:**

CDDD11-8 is an orally bioactive pharmacological inhibitor of CDK9. In AML cells, CDDD11-8 suppressed the proliferation and caused robust inhibition of tumor growth in vivo via synergistic inhibition of FLT3. The drug candidate has potential to overcome cancer cell resistance to FLT3 inhibition by concurrently blocking the CDK9-mediated upregulation of cancer cell-survival genes.

**Abstract:**

Mutations in FMS-like tyrosine kinase 3 (FLT3) occur in approximately one-third of AML patients and are associated with a particularly poor prognosis. The most common mutation, FLT3-ITD, is a self-activating internal tandem duplication (ITD) in the FLT3 juxtamembrane domain. Many FLT3 inhibitors have shown encouraging results in clinical trials, but the rapid emergence of resistance has severely limited sustainable efficacy. Co-targeting of CDK9 and FLT3 is a promising two-pronged strategy to overcome resistance as the former plays a role in the transcription of cancer cell-survival genes. Most prominently, MCL-1 is known to be associated with AML tumorigenesis and drug resistance and can be down-regulated by CDK9 inhibition. We have developed CDDD11-8 as a potent CDK9 inhibitor co-targeting FLT3-ITD with *K*_i_ values of 8 and 13 nM, respectively. The kinome selectivity has been confirmed when the compound was tested in a panel of 369 human kinases. CDDD11-8 displayed antiproliferative activity against leukemia cell lines, and particularly potent effects were observed against MV4-11 and MOLM-13 cells, which are known to harbor the FLT3-ITD mutation and mixed lineage leukemia (MLL) fusion proteins. The mode of action was consistent with inhibition of CDK9 and FLT3-ITD. Most importantly, CDDD11-8 caused a robust tumor growth inhibition by oral administration in animal xenografts. At 125 mg/kg, CDDD11-8 induced tumor regression, and this was translated to an improved survival of animals. The study demonstrates the potential of CDDD11-8 towards the future development of a novel AML treatment.

## 1. Introduction

Kinase inhibition, as a molecularly targeted therapy, has become a rapidly growing field in oncology due to an ever-increasing understanding of the role of kinases in pivotal oncogenic pathways. Although the initial efforts were mainly focused on targeting receptor tyrosine kinases (RTKs), recent expansion into the inhibition of basal cellular processes disproportionately exploited by cancers is garnering momentum [1]. One of the hallmarks of cancer cell transformation is the accumulation of gene mutations leading to the simultaneous activation of multiple pathways involved in proliferation and/or survival. The concurrent targeting of these pathways has been explored previously, and there is a strong indication that these strategies can lead to the development of effective anticancer therapeutics [2,3].

The *FLT3* gene encodes a tyrosine kinase receptor that plays a key role in the proliferation, differentiation, and survival of hematopoietic cells by activating downstream signaling pathways, including the PI3K and RAS pathways [4,5]. Gain-of-function mutations of FLT3 by internal tandem duplication (ITD) in the juxtamembrane domain or point mutations in its tyrosine kinase domains (FLT3-TKD) were found in approximately 30 to 40% of AML patients, engendering poor prognostic outcomes [4,6]. These constitutively active variants of FLT3 drive the sustained proliferation of early hematopoietic cells [4,6]. Several small-molecule FLT3 inhibitors have been evaluated clinically, and gilteritinib was the first FDA-approved FLT3 inhibitor to have been launched in the US for the treatment of relapsed/refractory FLT3-mutant AML. However, the overall clinical responses with these targeted inhibitors have been found to be transient due to the eventual development of primary or acquired resistance [7].

Cyclin-dependent kinases (CDKs) play key roles in the regulation of various cellular processes, including division (e.g., CDKs 1, 2, 4, and 6) and transcription (e.g., CDKs 7–13) [8,9,10,11,12,13,14]. CDK9-cyclin T facilitates the productive elongation of transcripts by two mechanisms: (1) phosphorylation of the carboxyl-terminal domain (CTD) of RNA polymerase II (RNAP II), which serves as a recruiting site for factors necessary for transcriptional elongation, and (2) phosphorylation of the negative elongation factor (NELF) and 5,6-dichloro-1-β-*D*-ribofuranosylbenzimidazole sensitivity inducing factor (DSIF), thereby providing relief from their induced pausing of RNAP II-mediated elongation of transcripts [15,16,17,18]. The overactivation of CDK9 in cancer cells drives the constant production of short-lived proto-oncogenes and anti-apoptotic proteins (e.g., MYC, MCL-1, and XIAP) that are important contributors to neoplastic transformation [19,20]. The persistent demand for CDK9 renders cancer cells highly vulnerable to its inhibition when compared to non-transformed cells [21]. This has been well described in AML, where CDK9 is required for the upregulated transcription of both anti-apoptotic (e.g., MCL-1) and cell cycle regulatory (e.g., cyclin D) proteins [22,23,24]. In addition, chromosomally rearranged mixed lineage leukemia (*MLL*) gene fusions depend on CDK9 for increased mRNA transcription and subsequent production of oncogenic proteins (e.g., HOX and FLT3), leading to reduced differentiation and enhanced survival of leukemic blast cells [25,26,27]. Over the years, many CDK9 inhibitors have been identified and some have progressed to clinical trials. However, most of the early generation inhibitors (e.g., flavopiridol, dinaciclib) were non-selective and their clinical development is hindered due to off-target toxicities [28]. Recently, a new class of more selective CDK9 inhibitors have emerged (e.g., BAY1143572 and AZD4573) and they are being studied in phase I clinical trials [29,30].

In this study, we report on the biochemical and pharmacological characteristics of CDDD11-8, a novel and highly selective inhibitor of CDK9 and FLT3. CDDD11-8 reduced the proliferation of leukemia cell lines and was particularly effective against those harboring FLT3-ITD mutation. It demonstrated potent anti-tumor efficacy in an AML tumor xenograft model following oral administration. Overall, the approach of inhibiting the transcription of genes important for the survival of leukemic cells through the co-targeting of CDK9 and FLT3-ITD offers a new avenue for addressing the clinical limitations currently faced with FLT3-ITD monotherapy.

## 2. Materials and Methods

**Figure 1:**
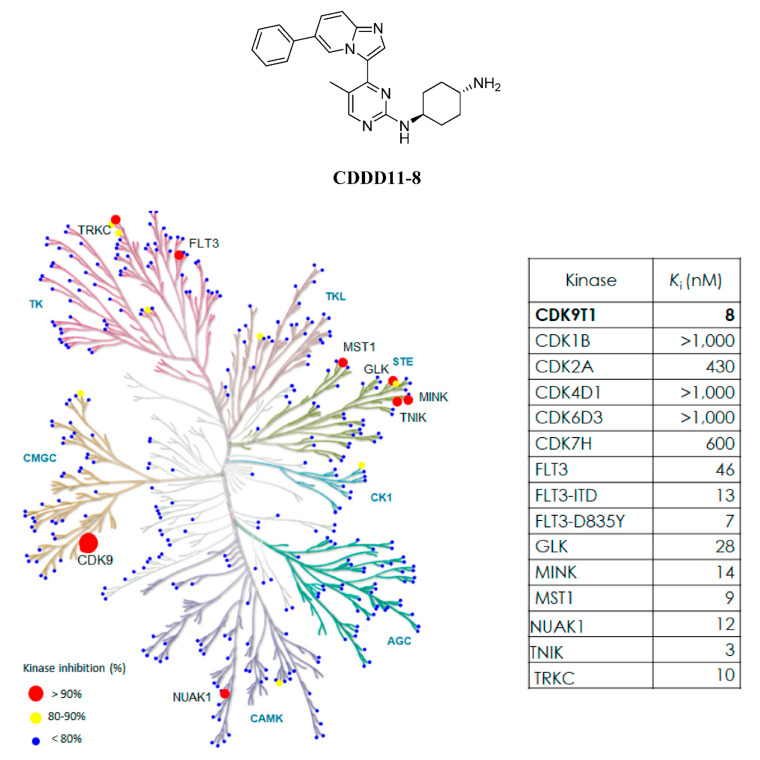
**Structure and kinase inhibition of CDDD11-8**. A kinome-wide selectivity test was carried out against 369 human kinases using 1 µM (~120 × CDK9 *K*_i_ value) compound concentration. All data were generated with radioactive assays by Reaction Biology (Malvern, PA, USA). Red, yellow, and green dots represent >90%, 80–90%, and <80% inhibition of the kinase, respectively. IC_50_ values were determined from concentration–response experiments and the apparent inhibition constants (*K*_i_) were calculated from IC_50_ values using the Cheng–Prusoff equation [32].

**Compounds and reagents**. CDDD11-8 (Figure 1) was synthesized in the laboratory of Drug Discovery and Development (Adelaide, SA, University of South Australia, Australia) according to the method described previously (Wang 2018). Dimethyl sulfoxide (DMSO) and resazurin were purchased from Sigma-Aldrich (Castle Hill, NSW, Australia).

**Kinase assay**. The ADP Glo^TM^ kinase assay (Promega, Madison, WI, USA) was used to assess the inhibition by CDDD11-8 of CDKs 1, 2, 4, 6, 7, and 9, as well as the mutant isoforms of FLT3. The assays were performed in accord with the manufacturer’s protocol (Promega) as described previously [31]. Briefly, serial three-fold dilutions of CDDD11-8 were prepared in 100% DMSO from a 2 mM stock solution, followed by 1:40 dilution into Milli-Q water. Subsequently, 1 µL of each sample was incubated with 4 µL of kinase mixture containing a particular CDK enzyme and its substrate, standard kinase buffer [3 mM MgCl_2_, 50 mM HEPES-NaOH pH 7.5, 3 mM MnCl_2_, 3 µM Na-orthovanadate, 1 mM dithiothreitol (DTT)], kinase dilution buffer (containing 0.25 mg/mL PEG20,000, 50 mM HEPES-NaOH pH 7.5, 1 mM DTT), and ATP at 37 °C for 30 to 40 min. The final reaction mixture contained 0.5% DMSO in all samples, and the applied kinase concentrations were 1, 1, 10, 40, 20, and 50 nM for CDK1, 2, 4, 6, 7, and 9, respectively. The reaction was stopped by adding ADP-Glo reagent and the mixture was incubated further in the dark at room temperature for 40 min. As a final step, kinase detection reagent was added to each mixture and incubated at room temperature for 30 to 60 min. Luminescence was read using an EnVision^®^ multilabel plate reader (PerkinElmer, Beaconsfield, UK), and half-maximum inhibitory concentrations (IC_50_) were calculated using a non-linear regression model with a variable slope, plotting log (inhibitor concentration) vs. response using the following formula:

$$A_{r}\left(\%\right)=\mathrm{Bottom}+\frac{\left(\mathrm{Top}-\mathrm{Bottom}\right)}{1+{\left(\frac{\mathrm{Inhibitor}\;\mathrm{Concentration}}{{\mathrm{IC}}_{50}}\right)}^{\mathrm{HillSlope}}}$$

where the relative activity A_r_ (%) is the signal relative to the controls, IC_50_ is the half maximum concentration, Hillslope is the steepness of the dose-response curve, Top and Bottom are plateaus reflecting the maximum and minimum in responses on the *Y*-axis (GraphPad Prism version 7, San Diego, CA, USA). *K*_i_ values were calculated from the IC_50_ using the Cheng−Prusoff equation [32]. Extensive specificity assessment was carried out by Reaction Biology Corporation (Malvern, PA, USA) with 369 kinases using 1 µM CDDD11-8 concentration in a Kinase Hotspot radiolabel assay (for experimental details, please see (Reaction Biology https://www.reactionbiology.com/). Subsequently, *K*_i_ values were determined for kinases with greater than 90% inhibition.

**Cell culture**. MV4-11, HL60, K-562, THP-1, MO91, and U937 cell lines were from the ATCC (Rockville, MD, USA); MOLM-13, NB4, and PL21 cell lines were from the DSMZ (Braunschweig, Germany); and Jurkat cells were from the ECACC (Salisbury, UK). All cells were cultured in RPMI medium (Sigma-Aldrich) supplemented with 10% to 20% of heat-inactivated fetal bovine serum (Sigma-Aldrich), and cells were maintained at 37 °C, 5% CO_2_, and 95% humidity. All the cell lines were tested to be mycoplasma-negative using MycoAlert^TM^ mycoplasma detection kit (Lonza, Derrimut, VIC, Australia).

**Cell viability assay**. The viability of leukemia cell lines after incubation with CDDD11-8 was determined by their ability to reduce resazurin, as described previously [3,33]. Cells (5 × 10^3^) were seeded into 96-well plates (Corning Inc., Corning, NY, USA) and incubated overnight at 37 °C. Subsequently, they were incubated with CDDD11-8 (concentrations ranging from 0.003 µM to 31.6 µM; using three-fold serial dilutions) or DMSO (final concentration of 0.1%) for 72 h. Afterwards, cells were incubated with 20 µL of resazurin (0.1 mg/mL in PBS) for 4 h at 37 °C under 5% CO_2_, and the fluorescence intensities were measured using an EnVision^®^ multilabel plate reader at 570 nm (excitation)/585 nm (emission). The concentration required to inhibit cellular growth by 50% (GI_50_) was determined using the same non-linear regression model as shown above, with A_r_ (%) = % cell viability, and IC_50_ replaced with GI_50_.

**Detection of apoptosis and cell cycle analysis**. Detection of apoptosis and cell cycle analysis were performed as described previously [3,34]. Cells (1 × 10^5^ cells/mL) were seeded into a six-well plate (Corning) and incubated overnight at 37 °C, 5% CO_2_ prior to incubation with different concentrations of CDDD11-8 or control (0.1% final DMSO concentration) for 6, 24, or 72 h. For washout experiments, cells were incubated with compounds for 6 h, then washed three times with 1 × PBS interspersed with centrifugation at 300× *g* for 5 min to remove compound. After incubation at 37ºC under 5% CO_2_, cells were centrifuged at 300× *g* for 5 min to form a pellet, washed with PBS, and incubated with Annexin V/PI (Becton Dickenson, Franklin Lakes, NJ, USA) for 15 min in the dark. For cell cycle analysis, cells were fixed with 70% ethanol (*v*/*v*, 4 °C) and incubated in the dark with cell cycle staining solution (50 µg/mL PI, 0.1 mg/mL RNase A, 0.05% Triton X-100) for 1.5 h. Samples were analyzed using a CytoFlex flow cytometer (Beckman Coulter, Brea, CA, USA). Data were analyzed with CytExpert software version 2.1 (Beckman Coulter, Brea, CA, USA).

**Western blotting**. Western blotting was performed as described previously [24,35]. Briefly, cells were lysed after incubation with different concentrations of compounds or control (0.1% DMSO) at the indicated times, samples were denatured at 95 °C for 5 min, and proteins having different molecular weights within each sample were separated by electrophoresis (Bio-Rad, Hercules, CA, USA) on polyacrylamide gels (4–20%, Bio-Rad). Resolved proteins were electrophoretically transferred onto a PVDF membrane and blocked by incubating with 10% *w/v* non-fat powdered milk dissolved in 1 × Tris-buffered saline-tween 20 (TBST) for 1 h at room temperature prior to incubating overnight with primary antibodies at 4 °C with slight shaking. Chemiluminescence was detected with a ChemiDoc^TM^ gel imaging system after the membranes have been exposed to ECL substrates (GE Healthcare, PA, USA), and the Image Lab software (Bio-Rad) was used for image capture. The primary antibodies used were: β-Actin (Invitrogen, Cat MA515739, 1:5000), β-Tubulin (Cell Signaling Technology, Cat 2146, 1:1000), AKT (Cell Signaling Technology, Cat 9272, 1:1000), c-MYC (Cell Signaling Technology, Cat 5605, 1:1000), Caspase-3 (Cell Signaling Technology, Cat 9665, 1:1000), Cleaved PARP (Cell Signaling Technology, Cat 5625, 1:1000), ERK1/2 (p44/42 MAPK, Cell Signaling Technology, Cat 4695, 1:1000), GAPDH (Invitrogen, Cat PA1987, 1:1000), MCL-1 (Cell Signaling Technology, Cat 942965, 1:1000), PARP (Cell Signaling Technology, Cat 9532, 1:1000), Phospho-AKT (Ser473) (Cell Signaling Technology, Cat 9271, 1:1000), Phospho-ERK (Thr202/Tyr204) (Cell Signaling Technology, Cat 4370, 1:1000), Phospho-FLT3 (Tyr591) (Cell Signaling Technology, Cat 3461, 1:1000), Phospho-STAT5 (Tyr694) (Cell Signaling Technology, Cat 9359, 1:1000), RNAP II CTD (BioLegend (CA, USA), Cat 664906, 1:1000), RNAP II CTD Phospho-Ser2 (BioLegend, Cat 920204, 1:1000), RNAP II CTD Phospho-Ser5 (Abcam, Cat ab5408, 1:1000), STAT5 (Cell Signaling Technology, Cat 9363, 1:1000), and XIAP (Cell Signaling Technology, Cat 14334, 1:1000). Bands were quantified using ImageJ software (US National Institutes of Health, Bethesda, MD, USA).

**Animal experiments**. All procedures conformed with the ethical principles described in the Australian Code for the Care and Use of Animals for Scientific Purposes, 8th edition (2013), and all experiments have been approved by the Animal Research Ethics Committee of the University of South Australia (Animal Ethics Numbers: U05-18, U14-17, U21-16). BALB/c nude (nu/nu) and hairy mice (6–8 weeks old, male and female) were obtained from the Animal Resource Centre (ARC, Perth, WA, Australia), and the Nude Breeding Colony was maintained by the Core Animal Facility, University of South Australia. All animals were acclimatized for one week to the Core Animal Facility in a pathogen-free room with 12-h day and 12-h night cycles, including a 30-min dawn/dusk cycle, at 22 ± 1 °C and 55% humidity. Autoclaved high fat chow supplied by Specialty Feeds (Western Australia, Australia) and acidified water (pH 3.2) were provided *ad libitum*. Groups of five mice were held in individually ventilated mouse cages (Tecniplast Green Line, West Chester, PA, USA), and bedding (1/8-inch corn cob bedding, from Corn-cob-ology Pty Ltd., Mt. Kuring gai, Sydney, NSW, Australia) with shredded paper and/or tissues was changed every week.

**Pharmacokinetics**. The pharmacokinetics of CDDD11-8 was determined in mice after single oral (PO) and intravenous (IV) doses. CDDD11-8 was administered at a dose of 10 or 100 mg/kg PO or 2 mg/kg IV to BALB/c hairy mice (*n* = 10 per route of administration; both male and female). The IV dose consisted of CDDD11-8 dissolved in 10/90 N-methyl-2-pyrrolidone (NMP)/acetate buffer (pH 4.5), while the PO doses of CDDD11-8 were dissolved in acetate buffer at pH 4.74. At nominal times, up to three blood samples were collected from individual mice at non-consecutive times from the right cheek, left cheek, and heart of an individual mouse. The concentrations in these samples were measured by validated LC-MS/MS methods (limit of quantification = 5 ng/mL). Pharmacokinetic parameters were estimated after fitting compartmental models to the concentration–time data (Phoenix, Certara, NJ, USA). Specifically, a one-compartmental kinetic model with first-order input was used after an oral dose, and a two-compartmental model was fitted to the IV data. Acceptability of models was evaluated from values for Akaike Information Criterion (AIC), Schwarz’s Bayesian Criterion (SIB), and % Coefficient of Variance (CV) for parameters, where lower values were indicative of a better fit. Other considerations were variances in the concentrations of quality control samples and visual inspection of model fit. Oral bioavailability (F) was calculated from values of AUC after PO and IV administration after correcting for the differences in doses.

***In vivo* xenograft study**. The *in vivo* efficacy of CDDD11-8 on tumors developed in mice from AML cells was evaluated by engrafting 5 × 10^6^ MV4-11 cells suspended in a 1:1 mixture of Matrigel^TM^ (Becton Dickinson, Corning, NY, USA) and serum-free medium (100 µL) subcutaneously into the right flank of female BALB/c nude (nu/nu) mice (6–7 weeks). When the tumor reached a volume of 100–200 mm^3^, mice were randomized into 3 groups (*n* = 7). Mice in G1 received vehicle (acetate buffer at pH 4.74), while mice in G2 and G3 received CDDD11-8, at 75 and 125 mg/kg, respectively. Both CDDD11-8 and vehicle were administered orally, once a day for 28 days. Clinical signs of toxicity were observed daily; body weights were measured daily during the dosing period and every other day thereafter. Tumor dimensions, i.e., length (L) and width (W), were measured every other day using Vernier calipers. The tumor volume was calculated using the formula 0.52 × L × W^2^, where L and W are perpendicularly measured diameters and L ≥ W. Mice were humanely killed by CO_2_ asphyxiation when the tumor volume reached 1500 mm^3^ or the percent loss of body weight exceeded 15%, or the study was completed.

Data were analyzed statistically using GraphPad Prism version 7. Descriptive statistics (frequency, mean, percentage) were used to report tumor volume, body weights, daily change in body weights from day 1 of dosing, and the ratio of tumor volume between treatment and control groups (T/C). Statistical differences in tumor volume between treated and control groups were analyzed using two-way ANOVA, where days on which the tumor volume was measured and treatments were the two independent factors, with a two-tailed significance cut-off level of *p* < 0.05. Tumor growth inhibition (TGI) was calculated as %TGI = [((VCD − VCB) − (VTD − VTB))/(VCD − VCB)] × 100, where VCD and VTD were the tumor volumes of the vehicle and treated groups on the calculated day, respectively, and VCB and VTB were the tumor volumes of the vehicle and treated groups at baseline (day 1). Kaplan–Meier survival analysis was performed, and group comparisons were done with the Gehan–Breslow–Wilcoxon test with Bonferroni correction method.

## 3. Results

**CDDD11-8 is a potent inhibitor of CDK9 and FLT3**. The compound was identified through a medicinal chemistry-driven hit-to-lead optimization program (Wang et al., 2018). The ADP-Glo method was used to test novel compounds for their inhibitory activities against CDKs1, 2, 4, 6, 7, and 9, as well as FLT3. CDDD11-8 was found to inhibit CDK9 and FLT3-ITD with *K*_i_ values of 8 and 13 nM, respectively, whilst there was 75- to 328-fold lower potency for other members of the CDK family. The kinome selectivity of CDDD11-8 was further investigated against a panel of 369 human kinases (Reaction Biology Corporation, Malvern, PA, USA) using an assay format with radiolabeled ATP and 1 µM compound concentration (>120 × *K*_i_ of CDK9). Only seven kinase families, i.e., TRKC, FLT3, NUAK1, GLK, MINK, TNIK, and MST1, were inhibited potently (Supplementary Table S1), with *K*_i_ values in the range of 10 to 46 nM (Figure 1).

**Anti-proliferative effects of CDDD11-8 on leukemic cells**. Further testing revealed leukemic cells that harbored the FLT3-ITD mutation and MLL fusions, such as MV4-11 (FLT3^ITD/ITD^/MLL-AF4) and MOLM-13 (FLT3^ITD/WT^/MLL-AF9), and those driven by TRK fusions (e.g., MO91) to be the most sensitive to the growth inhibitory effects of CDDD11-8 (GI_50_ < 0.10 µM, Table 1). Cell lines with either an MLL fusion only or FLT3-ITD mutation only were less sensitive to CDDD11-8, with GI_50_ values of 0.46 µM and 0.34 µM for THP-1 (FLT3^WT/WT^/MLL-AF9) and PL21 (FLT3^ITD/WT^) cells, respectively.

**Figure 2:**
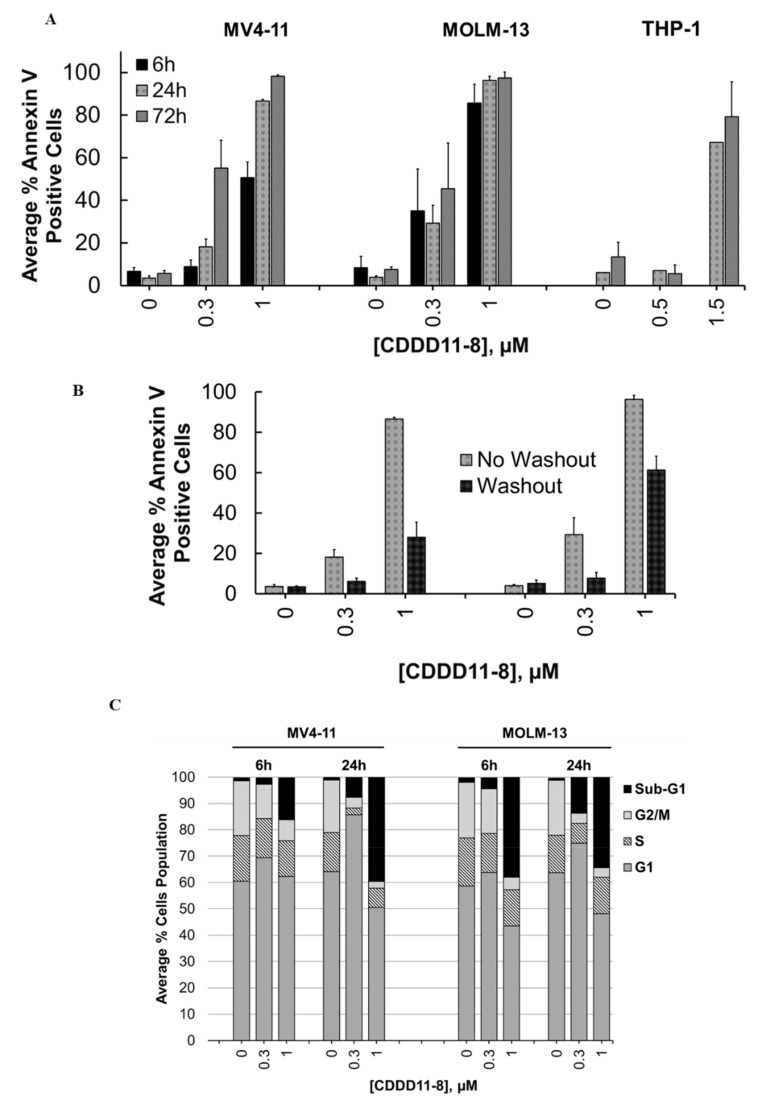
**Induction of apoptosis and cell cycle effect of CDDD11-8 on leukemia cell lines**. (**A**) MV4-11, MOLM-13, and THP-1 cells were incubated with CDDD11-8 using the concentrations and incubation times as indicated. Induction of early apoptosis was monitored using flow cytometry with Annexin V staining. (**B**) Comparison of Annexin V-positive MV4-11 and MOLM-13 cells incubated with CDDD11-8 for 24 h (no washout) or 6 h followed by a 24 h drug-free period (washout). (**C**) MV4-11 and MOLM-13 cells were incubated with 0.3 and 1 µM CDDD11-8 for 6 or 24 h, and their cell cycle progression was investigated using flow cytometry with fixed- and PI-stained cells. Data for (**A**,**B**) represent the mean ± SD values of two independent experiments. Data for (**C**) represent the average values of at least two independent experimental replicates.

In the next step, we performed a comparative analysis of CDDD11-8 mediated apoptotic effects in MV4-11, MOLM-13, and THP-1 cells using the annexin V/PI assay. The extent of apoptosis was dose- and time-dependent as indicated by the percentage of Annexin V-positive cells (Figure 2A, Supplementary Figures S1–S6). MV4-11 and MOLM-13 cells appeared to be more sensitive to the induction of apoptosis. Incubation with 1 µM CDDD11-8 for 6 h triggered apoptosis in 51% and 86% of the MV4-11 and MOLM-13 cells, respectively (Figure 2A, Supplementary Figures S1–S6). In contrast, there was less impact on the THP-1 cells, where the induction of apoptosis was observed at a higher concentration (1.5 µM) and extended incubation times (24 and 72 h) (Figure 2A).

The reversibility of apoptotic effects in the MV4-11 and MOLM-13 cells was investigated with compound washout after 6 h of incubation, followed by an additional 24 h of incubation without a compound. The numbers of apoptotic cells were only marginally reduced at a concentration of 1 µM CDDD11-8, with 28% and 61% apoptotic MV4-11 and MOLM-13 cells, respectively (Figure 2B, Supplementary Figures S7 and S8).

The effects of CDDD11-8 on the cell cycle were investigated by incubating MV4-11 and MOLM-13 cells with 0.3 and 1 µM for 6 or 24 h (Figure 2C, Supplementary Figures S9–S12). Both cell lines showed a very strong increase in the sub-G_1_ populations, indicating cell death. Notably, the sub-G_1_ cell population of both the MV4-11 and MOLM-13 cells increased by greater than 40-fold after 24 h of incubation with a 1 µM compound (Figure 2C, Supplementary Figures S9–S12). A slight trend towards an increase in the G_1_ population of the MV4-11 cells (16% increment) was noted after incubation with 0.3 µM CDDD11-8 for 24 h (Figure 2C).

**Figure 3:**
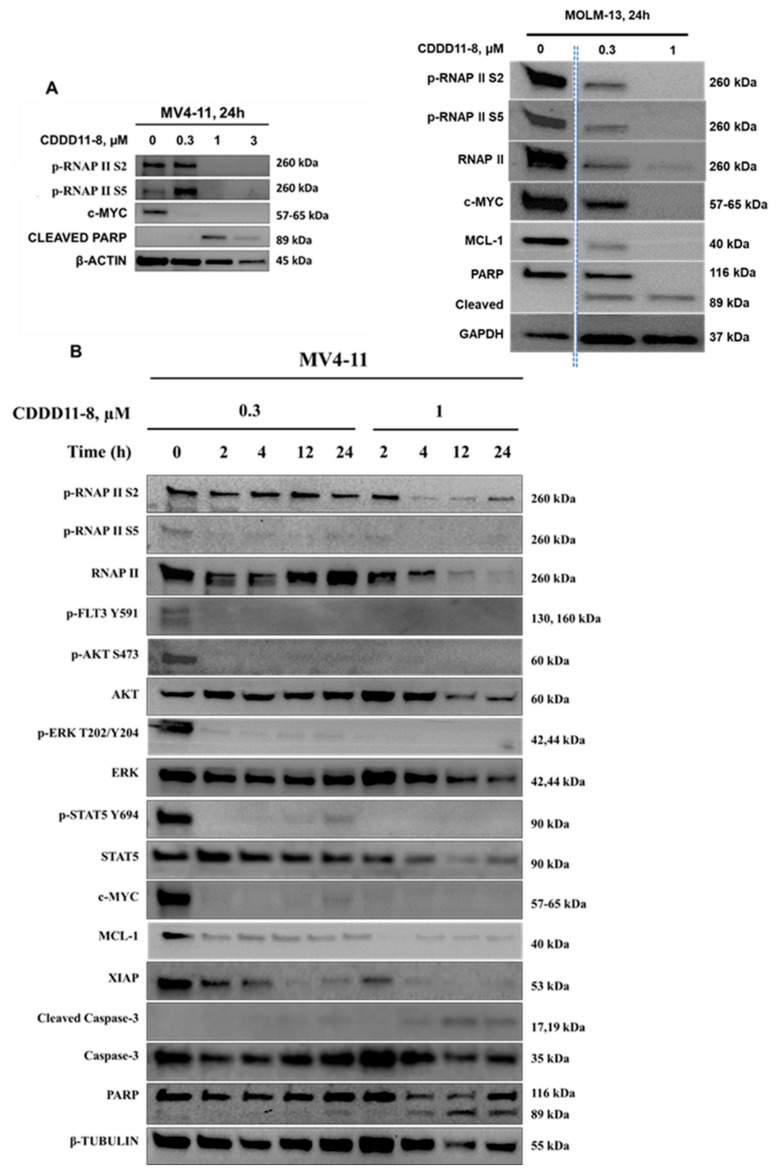
**Effect of CDDD11-8 on cellular signaling pathways in leukemia cell lines.** (**A**) Western blot analysis of lysates prepared from MV4-11 and MOLM-13 cells incubated with increasing concentrations of CDDD11-8 for 24 h. (**B**) MV4-11 cells were analyzed by western blot after being incubated with concentrations of 0.3 and 1 µM for the indicated times. β-Actin, GAPDH, or β-Tubulin were used as loading controls. Representative blots of two independent experiments are shown. For MOLM13 cells, the dotted lines indicate that the images were cropped from nonadjacent wells of the same gel.

**Effect of CDDD11-8 on cellular signaling pathways**. The molecular mechanisms of CDDD11-8 were further explored by examining the downstream signaling pathways of CDK9 and FLT3. CDDD11-8 suppressed the phosphorylation of RNAP II CTD in a concentration-dependent manner at Ser2 (p-RNAP II Ser2) and Ser5 (p-RNAP II Ser5) in MV4-11 and MOLM-13 cells, confirming cellular CDK9 inhibition (Figure 3A,B; Supplementary Figures S13–S15). The cell lines MV4-11 and MOLM-13 were very sensitive to CDDD11-8, likely due to the presence of CDK9-dependent MLL fusion mutations.

In a time-course study with MV4-11 cells, CDDD11-8 reduced the levels of p-RNAP II Ser2 and p-RNAP II Ser5 by approximately 20% within 2 h, and these levels declined progressively until 24 h (Figure 3B). Longer incubation of MV4-11 cells with a higher concentration of CDDD11-8 (1 µM) decreased the level of total RNAP II and tubulin, which might be ascribed to a significant number of cells undergoing apoptosis (Figure 3B).

Next, the effects of CDDD11-8 on FLT3 were assessed in MV4-11 cells. FLT3 mutations are known to drive sustained proliferation by activating downstream PI3K-AKT-mTOR or RAS-RAF-ERK pathways [7]. CDDD11-8 reduced the level of p-FLT3 Tyr591, a primary marker of FLT3-ITD signaling, leading to a reduction in the phosphorylation of its downstream targets p-STAT5 Tyr694, p-AKT Ser473, and p-ERK Thr202/Tyr204 (Figure 3B).

CDDD11-8 suppressed the expressions of c-MYC, MCL-1, and XIAP in MV4-11 and MOLM-13 cells (Figure 3A,B). These suppressions were virtually complete in all three cell lines at the highest concentration used (Figure 3A,B). The expression of c-MYC in MV4-11 cells was completely blocked as early as 2 h after incubation with 0.3 and 1 µM CDDD11-8 (Figure 3B). Meanwhile, the expressions of MCL-1 and XIAP were reduced to almost undetectable levels following 24 h of incubation at 1 µM (Figure 3B). The apoptotic mechanism of CDDD11-8 was linked with cleavage of caspase 3 (observed in MV4-11 cells) and PARP (noted in both cell lines) (Figure 3A,B). Overall, these results indicated that CDDD11-8 is most likely to induce apoptosis in the leukemia cell lines by modulating the cellular activity of CDK9 and FLT3.

**Pharmacokinetic studies in mice**. Profiles of the concentrations of CDDD11-8 in mice after IV (2 mg/kg) and oral doses (10 and 100 mg/kg) are shown in Supplementary Table S2 and Figure S16. The pharmacokinetic data are presented in Table 2. The blood to plasma concentration ratio (B/P) of CDDD11-8 was 0.71 (Table 3). Accordingly, the blood clearance of CDDD11-8 in mice (i.e., plasma clearance divided by B/P) was calculated to be 3.1 L/h.kg, and its volume of distribution was 1.71 L/kg. Its oral bioavailability after a single dose of 10 mg/kg was 30%. Its plasma exposure after a 100 mg/kg PO dose was found to be nearly nineteen-times greater than the exposure after 10 mg/kg dose (3.4 vs. 64 µM·h, respectively), and the calculated bioavailability value was 56% (Table 2).

**Figure 4:**
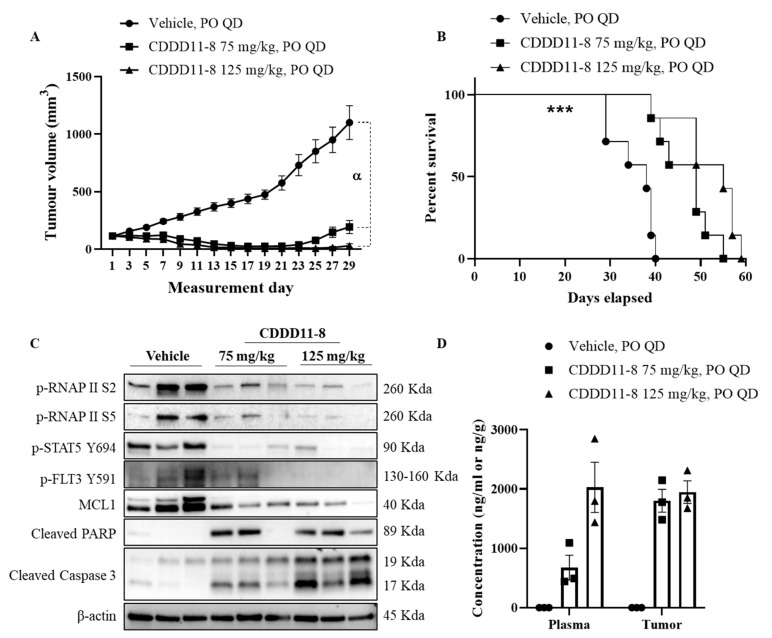
***In vivo* efficacy of CDDD11-8 against MV4-11 tumor xenografts.** MV4-11 tumor-bearing mice (*n* = 7) were treated orally with vehicle, CDDD11-8 (75 or 125 mg/kg), daily for 28 days. (**A**) Mean tumor volumes on across dosing periods. (**B**) Kaplan–Meier survival analysis. Another group of MV4-11 tumor-bearing mice (*n* = 3) were treated with vehicle, CDDD11-8 at 75 or 125 mg/kg, for 5 days, and tumors were harvested at 4 h post final dose for (**C**) immunoblotting to study *in vivo* target engagement and (**D**) determining drug concentration in plasma and tumor. MST: median survival time. Error bars indicate SEM. *** *p* < 0.002, compared to vehicle treatment. α refers to *p* values on (Table 4).

***In vivo* efficacy of CDDD11-8 against AML xenografts in mice**. The maximum tolerated dose (MTD) of CDDD11-8 was determined in non-tumor bearing female BALB/c nude (nu/nu) mice (*n* = 3). There were no overt clinical signs of toxicity or significant weight loss (i.e., >15%) up to an oral dose of 150 mg/kg administered once daily for 7 days (Supplementary Figure S17). At higher doses (200 mg/kg), a weight loss greater than 15% was observed in one mouse after five days of treatment, while the rest experienced >15% body weight loss after seven days of dosing (Supplementary Figure S17). Our proposed maximum dose of CDDD11-8 was 150 mg/kg once daily for seven days. For a subsequent *in vivo* efficacy study, we used the highest dose for CDDD11-8 (125 mg/kg), slightly lower than its MTD (150 mg/kg), since the dosing regimen involved once daily dosing of the compound for 28 days. All the animals received 28 doses of vehicle or CDDD11-8 at 75 or 125 mg/kg strength. A statistically significant inhibition of tumor growth was achieved starting from day 7 and 9 for 125 and 75 mg/kg CDDD11-8, respectively, compared to the vehicle treatment (Figure 4A and Table 4). Most notably, at 75 mg/kg, CDDD11-8 induced tumor regression on two out of seven animals starting on day 9, while, at 125 mg/kg, the tumors in all the mice started regressing from day 7. The tumor regression was maintained over the dosing period. On day 29, the tumors in the 75 mg/kg treated group had recommenced growth to variable degrees (Supplementary Table S3). However, the tumor regression was still maintained in six out of seven mice receiving CDDD11-8 at 125 mg/kg. On day 29, one and three mice from the 75 and 125 mg/kg CDDD11-8 treated groups, respectively, had unmeasurable tumor size (zero tumor volume). As a result, a T/C calculation revealed 15% and 1% on day 27, indicating a robust antitumor activity of the compound from both doses (Table 5). Although the antitumor efficacy of CDDD11-8 at 125 mg/kg was more potent than at 75 mg/kg (as shown by lower T/C by the former and higher tumor growth inhibition), the difference was not statistically significant, suggesting a further lowering of the dose might still produce potent antitumor efficacy (Table 4, Supplementary Table S4).

These tumor regressions and growth inhibitions were translated to a significant increase in the survival of the animals (*p* = 0.002) treated with 75 mg/kg (MST = 49 days) and 125 mg/kg (MST = 55 days) compared to the vehicle (MST = 38 days) (Figure 4B). This provided a 29% and 45% increase in the life span of the animals treated with 75 and 125 mg/kg CDDD11-8, respectively. Both doses of CDDD11-8 were well tolerated, and all the mice survived the full dosing regimen. There were no signs of clinical toxicity observed (Supplementary Figure S18).

To investigate the effect of CDDD11-8 on its targets *in vivo*, MV4-11 tumor-bearing mice (*n* = 3) were treated with the vehicle, CDDD11-8 (75 and 125 mg/kg), orally for 5 days. Four hours post final dose, tumor samples were collected and lysates prepared for western blot analysis (Figure 4C, Supplementary Figure S19). CDDD11-8 potently inhibited the phosphorylation of RNAPII at Ser2 and Ser5, confirming the inhibition of CDK9 *in vivo*. Phosphorylation of FLT3 at Tyr591 and STAT5 at Tyr 694 were also reduced by the CDDD11-8 treated groups, revealing effective inhibition of FLT3. These were accompanied by changes in the levels of the proteins involved in cancer cell survival. The level of short-half life antiapoptotic protein MCL1 was greatly reduced and correlates very well with potent activation of caspase 3 and cleavage of PARP, suggesting the occurrence of apoptosis *in vivo*, supporting the regression of tumors in mice treated with CDDD11-8.

We also determined the concentration of CDDD11-8 in the plasma and tumor (Figure 4D, Supplementary Table S5). When comparing the concentration of CDDD11-8 from 75 and 125 mg/kg, approximately similar levels of concentration were achieved in the tumor, whereas a three-times increase in the plasma concentration was detected after the higher dose (Supplementary Table S5). The tumor concentration from the 75 mg/kg dose was 2.5-times the plasma concentration. However, the plasma and tumor concentrations from 125 mg/kg were similar. Considering the concentration of CDDD11-8 in the tumor, its concentrations were 300-times the GI_50_ of CDDD11-8 against MV4-11 cells, suggesting that enough concentrations of the drug are available to induce robust antitumor efficacy.

## 4. Discussion

AML is a clinically and genetically heterogeneous disease that is characterized by the abnormal proliferation and differentiation of a population of myeloid stem cells [39]. Mutation in *FLT3* is one of the most common and frequent genetic aberrations in AML, making FLT3 an attractive therapeutic target, with several inhibitors in the development pipelines [7]. However, FLT3 inhibitors, when used as a monotherapy, have shown transient responses primarily due to drug resistance, which called for combined treatment approaches directed towards additional pathways of FLT3 signaling, such as PI3K/AKT and STAT5 [7,40,41]. In this context, the targeting of cellular proteins that function downstream of FLT3 (e.g., CDK9), which mediate the transcription of critical survival proteins (e.g., MCL-1) relevant to AML, might be a promising therapeutic strategy against the emerging resistance [22,24]. The present study describes the biological and pharmacological characterization of CDDD11-8, a novel inhibitor of CDK9 and FLT3 with potent anti-leukemic properties.

CDDD11-8 inhibited FLT3 and its mutated isoforms (e.g., FLT3-ITD and FLT3-D835Y). The targeting of these mutated kinases has significant clinical implications in some hematological malignancies. FLT3-ITD has been previously described as a driver mutation in AML, and the point mutation D835Y plays a crucial role in the development of resistance to FLT3-ITD inhibitors [5,7]. While AML patients with FLT3 mutation show a clinical response to inhibitors, it is not durable, and the disease progresses in almost all the patients. This is ascribed to the development of primary and secondary resistances, calling for combination treatment modalities to achieve a more sustained response [7]. Hence, co-targeting FLT3 and CDK9 provides a rational therapeutic approach for the following reasons: (i) the activity of both kinases is required for the induction and maintenance of acute leukemia [25,27], and (ii) the upregulated expression of MCL-1 (which is dependent on CDK9) is one of the mechanisms for the development of resistance to FLT3 inhibitors [7].

CDDD11-8 was a selective pharmacological inhibitor with > 50-fold more potency in its inhibition of CDK9 over other members of the CDK family (Figure 1). Such selectivity might be a valuable contributor to its overall safety as some CDKs (e.g., CDK1) are indispensable for the proliferation of normal cells and dose-limiting toxicities have been associated with pan-CDK inhibitors in clinical trials [42,43]. The CDK9-specific inhibitors BAY1143572 (IC_50_ = 6 nM) and ADZ4573 (IC_50_ < 4 nM) inhibited CDK9 with greater than 100- and 10-fold selectivity over other CDKs, respectively [29,30]. Here, caution must be exercised while making direct comparisons as these studies employed biochemical (kinase) assays having varying sensitivity. The dysregulation of the CDK9-mediated transcription of anti-apoptotic proteins (e.g., MCL-1) is a key mechanism employed by AML cells for their survival [22,24,44]. In addition, AML cells with MLL abnormalities are dependent on CDK9 to produce proteins that reduce the differentiation while enhancing the survival of cells (e.g., HOX, FLT3) [23,25,26]. Accordingly, CDDD11-8 inhibited the proliferation of leukemia cell lines with FLT3-ITD and MLL fusions (MV4-11 and MOLM-13 cells) more potently relative to cell lines lacking these mutations (e.g., U937) or those harboring either one of these genetic aberrations (e.g., PL21 and THP-1, Table 1).

A further kinome scan against 369 kinases revealed that, besides CDK9 and FLT3, CDDD11-8 also inhibited TRKC, NUAK1, GLK, MINK, TNIK, and MST1 potently. Among these kinases, the inhibition of TRKC is noteworthy as chromosomal rearrangement mutations involving *NTRK* genes lead to chimeric proteins with an oncogenic potential in some rare forms of AML and solid cancers [45]. In addition to this, some more of the above-mentioned kinases might also be relevant to cancer therapy. For example, the overexpression of NUAK1 has been linked to a poor prognosis in ovarian and colorectal cancers, and the oncogenic role of TNIK is deemed important in colorectal cancer [46,47,48]. However, with the aim of targeting FLT3-mutated AML with MLL fusions, we focused our investigations on the inhibitory activities against CDK9 and FLT3.

We showed that CDDD11-8 induced apoptosis in the MV4-11 and MOLM-13 leukemia cell lines harboring the clinically relevant genetic aberrations of MLL fusion and FLT3-ITD (Figure 2). MLL fusions and the FLT3-ITD mutation are known oncogenic drivers of acute leukemias and are dependent on the activity of CDK9 and FLT3, respectively [5,49]. Both cell lines, in comparison to THP-1 cells, showed strong activation of apoptosis, as indicated by increased annexin V signals. This early apoptosis marker was marginally stronger in the MOLM-13 cells, which might be due to the FLT3^WT/ITD^ genotype with one FLT3-ITD allele only. Hence, the proliferation of these cells might require additional oncogenic transformation, therefore relying on the stronger MLL fusion. This might explain the higher and earlier apoptosis observed in MOLM-13 cells compared to the homozygous FLT3-ITD MV4-11 cells. In contrast, MV4-11 cells showed more sub-G1 (dead cells) after 24 h of exposure to a 1 µM compound. CDDD11-8 inhibited the phosphorylation of RNAP II CTD at both Ser2 and Ser5 residues in MV4-11 and MOLM-13 cells and decreased the production of the anti-apoptotic MCL-1 and XIAP proteins (Figure 3). Furthermore, it inhibited the autophosphorylation of FLT3 in MV4-11 cells, which translated into a reduced phosphorylation of its downstream signaling targets, such as STAT5, AKT, and ERK. These cell lines are known to harbor both MLL fusion and FLT3-ITD mutations and thus are expected to be dependent on the activity of both CDK9 and FLT3. Here, it was noted that the reduction in the level of p-RNAP II Ser5 was comparable to or superior to that of p-RNAP II Ser2. Considering that only the latter is a fully established CDK9 phosphorylation residue, this effect might be explained by earlier observations that p-RNAP II Ser5 can be an additional phosphorylation target for CDK9 [50]. Furthermore, recent evidence showed that prolonged CDK9 inhibition, leading to the pausing of RNAP II at promoter-proximal sites, blocks a new round of transcriptional initiation, thereby limiting phosphorylation on Ser5 residues [51,52].

We conducted a series of oral dosage experiments to investigate the suitability of CDDD11-8 for this route of administration. If one assumes the liver as the major organ for the elimination of CDDD11-8, then a comparison of the blood clearance for CDDD11-8 (3.1 L/h.kg) with the hepatic blood flow in mice (i.e., 5.4 L/h.kg, (Boxenbaum, 1980)) would indicate an approximate extraction of CDDD11-8 by the liver of 57%. Furthermore, assuming that all of the administered dose dissolved completely in the gastrointestinal (GI) tract (soluble > 100 µM in water, Table 3) and that it would, therefore, be available for absorption, and, if there was minimal hindrance to its diffusion through the GI wall (partition co-efficient of 1.87), then, from its hepatic extraction, one would predict an oral bioavailability of about 43%. In contrast, the oral bioavailability of CDDD11-8, calculated based on AUC_0-∞_ values after IV and PO doses, was found to be around 30% (Table 2). This slightly lesser than predicted bioavailability value might be attributed to the efflux of CDDD11-8 out of enterocytes back into the GI lumen (efflux ratio = 4.9, Table 3), thereby reducing the amount of drug reaching the systemic circulation. In comparison to the total body water of 0.73 L/kg in mice, CDDD11-8 had a volume of distribution of 1.71 L/kg, which suggests a more extensive level of distribution outside plasma and/or blood. The exposure in the plasma after a 100 mg/kg PO dose was 19-times greater than exposure after a dose of 10 mg/kg (Table 2). This suggested that any limitation to the dissolution of CDDD11-8 in the intestinal lumen had not been reached across this ten-fold range of oral dose, but the two-times greater oral bioavailability might be attributed to some degree of saturation of active efflux and/or elimination during hepatic passage.

CDDD11-8 was highly efficacious in an MV4-11 AML xenograft model. The compound caused tumor regression and did eradicate the tumor totally in some animals (Figure 4, Supplementary Tables S3 and S4). The potent inhibition of tumor growth was translated into a significant increase in the life of animals treated with the compound. A pharmacodynamic study in tumor bearing mice has also shown that CDDD11-8 works by potently inhibiting CDK9 and FLT3, similar to the *in vitro* finding. The potent reduction of anti-apoptotic protein MCL1, coupled with the activation of caspase 3 and cleavage of PARP, substantiates the observation of tumor regression. From both doses of CDDD11-8, its concentration in the tumor tissue was approximately 300-fold higher than its GI_50_ against MV4-11 cells, suggesting that even lower doses of the compound could still induce robust antitumor activity (Supplementary Table S5).

## 5. Conclusions

Overall, this study suggests that CDDD11-8 is a promising therapeutic agent for the treatment of AML. Its inhibition of CDK9 with a concurrent reduction in the activity of FLT3 may offer an advantage in addressing the resistance and recurrence of AML. The drug possesses excellent biopharmaceutic and *in vivo* pharmacokinetic properties and can be developed in solid oral dosage forms for therapy. Given its potent antitumor efficacies and safety profile, further investigations of CDDD11-8 against a range of solid tumors associated with the CDK9-mediated transcription of oncogenes and pro-survival genes, i.e., MYC, MCL-1, Bcl-2, and XIAP, are also warranted.

## Figures and Tables

**Table 1:** Antiproliferative activities of CDDD11-8 against leukemia cell lines having different genetic backgrounds *.

Cell Line	Leukemia Type	Oncogenic Drivers	CDDD11-8 GI_50_ (µM ± SD)
MV4-11	AML	MLL-AF4, FLT3^ITD/ITD^	0.015 ± 0.004
MO91	AML	ETV6-NTRK3	0.032 ± 0.003
MOLM-13	AML	MLL-AF9, FLT3^ITD/WT^	0.070 ± 0.020
PL21	AML	K-RAS, FLT3^ITD/WT^	0.339 ± 0.034
THP-1	AML	MLL-AF9, FLT3^WT/WT^	0.459 ± 0.040
NB4	AML	PML-RARA	0.473 ± 0.107
HL60	AML	N-RAS	0.475 ± 0.025
JURKAT	ALL	FLT3 NULL	0.599 ± 0.090
U937	AML	MLLT10-PICALM	0.713 ± 0.093
K-562	CML	BCR-ABL	1.495 ± 0.047

* MLL-AF4 and MLL-AF9, a fusion of proteins formed due to t(4; 11) (q21; q23) and t(9; 11) (p22; q23) chromosomal rearrangement mutations, respectively; PML-RARA, a t(15; 17) (q22; q21) translocation mutation involving genes encoding PML and retinoic acid receptor alpha (RARA) [36]; MLLT10-PICALM is a fusion of MLLT10 and PICALM proteins as a result of t(10; 11) (p13; q14-21) chromosomal translocation mutation [37]; BCR-ABL, a Philadelphia chromosome formed by a reciprocal translocation between chromosome 9 (encoding abelson tyrosine kinase, ABL) and chromosome 22 (encoding breakpoint cluster region protein, BCR) [38]; GI_50_ values represent the mean ± standard deviation (SD) of at least two independent experimental replicates.

**Table 2:** Pharmacokinetic parameters of CDDD11-8 in mice.

Route	IV *	Oral *	
Dose (mg/kg)	2	10	100
C_max_ (ng/mL)	-	496 ± 108	3597 ± 822
t_max_ (h)	-	1.0 ± 0.1	2.0 ± 0.6
AUC (ng/mL/h)	906 ± 90	1364 ± 249	25,369 ± 3973
t_½_ (h)	1.8 ± 0.3	0.68 ± 0.30	6.8 ± 3.7
CL (mL/h/kg)	2208 ± 219	-	-
Vss (mL/kg)	1713 ± 246	-	-
F (%)	-	30	56

* Parameters (± standard error of the estimate, *n* = 10 per route of administration) were determined using compartmental analysis (Phoenix, Certara, NJ, USA).

**Table 3:** Biopharmaceutic properties of CDDD11-8.

Aqueous Solubility ^a^	μM	>100
Partition Coefficient ^b^	LogD_7.4_ (octanol)	1.87
Dissociation Constant ^c^	pKa	2.75, 4.95, 9.89
Caco-2 Permeability ^d^	A-B P_app_ (10^−6^ cm/s)	6.06 ± 0.49
	B-A P_app_ (10^−6^ cm/s)	29.5 ± 8.47
	Efflux ratio	4.87
Blood to plasma ratio	In mice	0.71

^a^ By turbidimetric method; ^b^ by shake flask assay; ^c^ by PH metric assay; ^d^ apparent permeability coefficient across the Caco-2 monolayer; efflux ratio = P_app B-A_/P_app A-B_ (Cyprotex Ltd., Macclesfield, UK).

**Table 4:** Statistical significance of the differences between the means of tumor volumes across dosing period.

Measurement Days	Comparison of Tumor Volume (*p*-Values) *		
	Vehicle vs. CDDD11-8 75 mg/kg	Vehicle vs. CDDD11-8 125 mg/kg	CDDD11-8 75 mg/kg vs. 125 mg/kg
1	0.9997	0.9994	0.9999
3	0.7745	0.62	0.9658
5	0.3783	0.1869	0.9064
7	0.0868	0.018	0.8129
9	0.0024	0.0001	0.7113
11	<0.0001	<0.0001	0.8224
13	<0.0001	<0.0001	0.8602
15	<0.0001	<0.0001	0.9495
17	<0.0001	<0.0001	0.9534
19	<0.0001	<0.0001	0.9462
21	<0.0001	<0.0001	0.9486
23	<0.0001	<0.0001	0.876
25	<0.0001	<0.0001	0.4531
27	<0.0001	<0.0001	0.0526

* Statistical differences in tumor volume between groups were analyzed using two-way ANOVA, where days on which the tumor volume was measured and treatments were the two independent factors, with a two-tailed significance cut-off level of *p* < 0.05.

**Table 5:** Percent of tumor volume in treatment group to tumor volume in vehicle treatment group *.

Measurement Days	T/C% at Dose of CDDD11-8 (mg/kg) *	
	75	125
1	101	102
3	75	66
5	60	47
7	50	36
9	32	16
11	22	12
13	12	4
15	8	3
17	6	2
19	5	2
21	5	2
23	5	1
25	9	1
27	15	1

* Percent T/C was calculated by dividing the average tumor volume of mice in CDDD11-8 treated group to average tumor volume of mice in vehicle treated group at a particular tumor measurement day.

## Data Availability

Data presented in this study are available on request from the corresponding author.

## Supplementary Materials

The following supporting information can be downloaded at: https://www.mdpi.com/article/10.3390/cancers14051113/s1, Table S1: Kinome-wide selectivity of CDDD11-8 at 1 µM, Table S2: Plasma concentrations of CDDD11-8 in mice after single PO (10 and 100 mg/kg) and IV (2 mg/kg) doses, Table S3: Tumor volume measurements throughout the treatment period, Table S4: Percent tumor growth inhibition throughout the treatment period, Table S5: Concentration of CDDD11-8 in plasma and tumor 4 h post final dose. Figure S1: Original flowcytometry data for Figure 2A (MV4-11, 6 h, AV), Figure S2: Original flowcytometry data for Figure 2A (MV4-11, 24 h, AV), Figure S3: Original flowcytometry data for Figure 2A (MV4-11, 72 h, AV), Figure S4: Original flowcytometry data for Figure 2A (MOLM-13, 6 h, AV), Figure S5: Original flowcytometry data for Figure 2A (MOLM-13, 24 h, AV), Figure S6: Original flowcytometry data for Figure 2A (MOLM-13, 72 h, AV), Figure S7: Original flowcytometry data for Figure 2B (MV4-11, 24 h washout, AV), Figure S8: Original flowcytometry data for Figure 2B (MOLM-13, 24 h washout, AV), Figure S9: Original flowcytometry data for Figure 2C (MV4-11, 6 h, cell cycle), Figure S10: Original flowcytometry data for Figure 2C (MV4-11, 24 h, cell cycle), Figure S11: Original flowcytometry data for Figure 2C (MOLM-13, 6 h, cell cycle), Figure S12: Original flowcytometry data for Figure 2C (MOLM-13, 24 h, cell cycle), Figure S13: Uncropped western blots for Figure 3A (MV4-11, 24 h, dose-range), Figure S14: Uncropped western blots for Figure 3A (MOLM-13, 24 h, dose-range), Figure S15: Uncropped western blots for Figure 3B (MV4-11, time-course), Figure S16: Plasma concentration-time profiles of CDDD11-8 in mice after single (A) IV dose of 2 mg/kg, PO doses of (B) 10 or (C) 100 mg/kg, Figure S17: Effect of CDDD11-8 on body weight of mice, Figure S18: Percent change in body weight of MV4-11 tumor-bearing BALB/c nude (nu/nu) mice, Figure S19: Uncropped western blots for Figure 4C (MV4-11 tumors).

## Nonstandard Abbreviations

AKT, Protein Kinase B; CDK, Cyclin-dependent Kinase; CL, Clearance; CTD, Carboxyl Terminal Domain; ERK, Extracellular Signal-regulated Kinase; GLK, Germinal Center Kinase-like Kinase; ITD, Internal Tandem Duplication; Ki, Inhibitory Constant; MCL-1, Myeloid Cell Leukemia 1; MINK, Misshapen/NIKs-related Kinase; MLL, Mixed Lineage Leukemia; MST1, Mammalian Ste20-like Kinase 1; mTOR, Mammalian Target of Rapamycin; NUAK1, Muscle-specific Knock-out of NUAK Family SNF1-like Kinase 1; PARP, Poly(ADP-ribose) Polymerase; PI, Propidium Iodide; PI3K, Phosphatidylinositol 3-Kinase; RNAP II, Ribonucleic Acid Polymerase II; RTK, Receptor Tyrosine Kinase; STAT5, Signal Transducer and Activator of Transcription 5; TKD, Tyrosine Kinase Domain; TNIK, Tumor Necrosis Factor Receptor-associated Factor 2 and Nck-interacting Kinase; TRKC, Tropomyosin Receptor Kinase C; XIAP, X-linked Inhibitor of Apoptosis Protein.
