# Supplementary material for: An Orally Bioavailable and Highly Efficacious Inhibitor of CDK9/FLT3 for the Treatment of Acute Myeloid Leukemia
Source: Cancers (Basel). 2022 Feb 22;14(5):1113. doi: 10.3390/cancers14051113 (PMC8909834; doi:10.3390/cancers14051113)
Supplement: Supplementary file 1 [file cancers-14-01113-s001.zip › cancers-1558513 - sup.pdf]

An orally bioavailable and highly efficacious CDK9/FLT3 inhibitor for the treatment of acute myeloid leukemia

Abel Tesfaye Anshabo, Laychiluh Bantie, Sarah Diab, Jimma Lenjisa, Alemwork Kebede, Gary Heinemann, Jasmine Karanjia, Benjamin Noll, Sunita K.C. Basnet, Manjun Li, Robert Milne, Hugo Albrecht, and Shudong Wang

Drug Discovery and Development, Clinical and Health Sciences, University of South Australia, Adelaide, SA 5000, Australia

Corresponding authors:

Shudong Wang

shudong.wang@unisa.edu.au

Tel. +61 8 8302 2372

Hugo Albrecht

hugo.albrecht@unisa.edu.au

Tel. +61 8 8302 1093

Drug Discovery and Development, Clinical and Health Sciences, University of South Australia, Adelaide, SA 5001, Australia

Keywords: CDK9, FLT3, Targeted Therapy, Cancer, Leukemia

Table S1: Kinome-wide selectivity of CDDD11-8 at 1 μM

Kinase	% Residual Activity	Kinase	% Residual Activity
ABL1	76.81	CDC7/DBF4	108.56
ABL2/ARG	67.47	CDK1/cyclin A	77.95
ACK1	64.45	CDK1/cyclin B	69.51
AKT1	95.00	CDK1/cyclin E	87.55
AKT2	115.95	CDK14/cyclin Y (PFTK1)	68.78
AKT3	77.24	CDK16/cyclin Y (PCTAIRE)	57.37
ALK	68.93	CDK17/cyclin Y (PCTK2)	34.10
ALK1/ACVRL1	96.94	CDK18/cyclin Y (PCTK3)	100.09
ALK2/ACVR1	120.83	CDK19/cyclin C	37.96
ALK3/BMPR1A	98.00	CDK2/cyclin A	55.03
ALK4/ACVR1B	95.33	CDK2/Cyclin A1	55.66
ALK5/TGFBR1	93.93	CDK2/cyclin E	55.63
ALK6/BMPR1B	104.85	CDK2/cyclin O	56.81
ARAF	91.51	CDK3/cyclin E	77.87
ARK5/NUAK1	7.93	CDK4/cyclin D1	89.29
ASK1/MAP3K5	90.16	CDK4/cyclin D3	79.70
Aurora A	79.26	CDK5/p25	77.79
Aurora B	89.62	CDK5/p35	84.47
Aurora C	82.08	CDK6/cyclin D1	80.68
AXL	44.44	CDK6/cyclin D3	96.61
BLK	47.19	CDK7/cyclin H	63.72
BMPR2	90.25	CDK9/cyclin K	6.40
BMX/ETK	71.22	CDK9/cyclin T1	18.48
BRAF	99.71	CDK9/cyclin T2	2.35
BRK	87.41	CHK1	95.24
BRSK1	97.41	CHK2	97.25
BRSK2	96.50	CK1a1	101.60
BTK	86.96	CK1a1L	89.97
CAMK1a	80.71	CK1d	17.64
CAMK1b	96.10	CK1epsilon	37.10
CAMK1d	87.06	CK1g1	62.78
CAMK1g	97.67	CK1g2	59.42
CAMK2a	20.26	CK1g3	63.19
CAMK2b	23.54	CK2a	80.32
CAMK2d	17.31	CK2a2	74.69
CAMK2g	96.80	c-Kit	97.48
CAMK4	97.29	CLK1	28.86
CAMKK1	76.50	CLK2	47.03
CAMKK2	47.73	CLK3	85.93

Kinase	% Residual Activity	Kinase	% Residual Activity
CLK4	26.13	ERK5/MAPK7	93.30
c-MER	52.32	ERK7/MAPK15	66.43
c-MET	102.69	ERN1/IRE1	41.82
COT1/MAP3K8	100.86	ERN2/IRE2	45.43
CSK	91.15	FAK/PTK2	63.27
c-Src	49.55	FER	69.96
CTK/MATK	123.43	FES/FPS	86.19
DAPK1	96.07	FGFR1	96.78
DAPK2	81.23	FGFR2	72.45
DCAMKL1	112.65	FGFR3	92.78
DCAMKL2	108.00	FGFR4	104.38
DDR1	63.07	FGR	73.44
DDR2	73.49	FLT1/VEGFR1	53.41
DLK/MAP3K12	45.82	FLT3	2.43
DMPK	96.16	FLT4/VEGFR3	23.30
DMPK2	26.33	FMS	72.98
DRAK1/STK17A	92.53	FRK/PTK5	97.14
DYRK1/DYRK1A	63.90	FYN	63.25
DYRK1B	22.24	GCK/MAP4K2	24.79
DYRK2	12.67	GLK/MAP4K3	9.75
DYRK3	23.24	GRK1	65.00
DYRK4	93.62	GRK2	95.92
EGFR	82.25	GRK3	91.90
EPHA1	93.09	GRK4	98.16
EPHA2	86.39	GRK5	88.87
EPHA3	94.03	GRK6	98.46
EPHA4	100.58	GRK7	70.73
EPHA5	83.79	GSK3a	70.11
EPHA6	88.37	GSK3b	94.46
EPHA7	104.57	Haspin	65.08
EPHA8	83.45	HCK	62.67
EPHB1	88.10	HGK/MAP4K4	32.80
EPHB2	80.25	HIPK1	105.33
EPHB3	92.67	HIPK2	104.03
EPHB4	80.97	HIPK3	124.78
ERBB2/HER2	101.28	HIPK4	52.40
ERBB4/HER4	96.91	HPK1/MAP4K1	35.41
ERK1	97.35	IGF1R	79.01
ERK2/MAPK1	108.63	IKKa/CHUK	98.45

Kinase	% Residual Activity	Kinase	% Residual Activity
IKKb/IKBKB	78.09	MEKK1	103.47
IKKe/IKBKE	91.65	MEKK2	105.32
IR	101.46	MEKK3	108.78
IRAK1	91.88	MEKK6	104.13
IRAK4	59.25	MELK	66.91
IRR/INSRR	100.54	MINK/MINK1	8.86
ITK	87.50	MKK4	75.60
JAK1	93.26	MKK6	95.69
JAK2	69.98	MKK7	95.38
JAK3	48.49	MLCK/MYLK	57.17
JNK1	105.02	MLCK2/MYLK2	25.93
JNK2	103.13	MLK1/MAP3K9	42.99
JNK3	81.35	MLK2/MAP3K10	76.47
KDR/VEGFR2	86.88	MLK3/MAP3K11	45.06
KHS/MAP4K5	20.31	MLK4	111.68
KSR1	92.34	MNK1	76.43
KSR2	100.78	MNK2	83.54
LATS1	57.37	MRCKa/CDC42BPA	97.68
LATS2	30.24	MRCKb/CDC42BPB	79.29
LCK	72.51	MSK1/RPS6KA5	61.57
LCK2/ICK	62.35	MSK2/RPS6KA4	97.43
LIMK1	12.95	MSSK1/STK23	90.76
LIMK2	93.75	MST1/STK4	8.89
LKB1	102.50	MST2/STK3	15.04
LOK/STK10	55.76	MST3/STK24	103.42
LRRK2	70.57	MST4	92.94
LYN	65.54	MUSK	72.72
LYN B	88.46	MYLK3	99.65
MAK	67.57	MYLK4	11.50
MAPKAPK2	105.62	MYO3A	87.29
MAPKAPK3	91.75	MYO3b	58.44
MAPKAPK5/PRAK	99.44	NEK1	40.75
MARK1	46.60	NEK11	100.34
MARK2/PAR-1Ba	23.09	NEK2	90.73
MARK3	36.99	NEK3	92.75
MARK4	22.42	NEK4	78.78
MEK1	98.87	NEK5	28.73
MEK2	79.06	NEK6	96.20
MEK3	71.48	NEK7	102.15
MEK5	99.58	NEK8	72.75

Kinase	% Residual Activity	Kinase	% Residual Activity
NEK9	84.70	PKCtheta	46.22
NIM1	89.88	PKCzeta	89.19
NLK	83.40	PKD2/PRKD2	62.69
OSR1/OXSR1	100.83	PKG1a	61.72
P38a/MAPK14	105.62	PKG1b	77.53
P38b/MAPK11	98.41	PKG2/PRKG2	87.04
P38d/MAPK13	99.02	PKN1/PRK1	63.80
P38g	100.22	PKN2/PRK2	88.31
p70S6K/RPS6KB1	94.48	PKN3/PRK3	81.00
p70S6Kb/RPS6KB2	98.61	PLK1	97.33
PAK1	105.64	PLK2	97.54
PAK2	96.71	PLK3	108.51
PAK3	87.48	PLK4/SAK	89.26
PAK4	99.68	PRKX	77.39
PAK5	86.17	PYK2	84.16
PAK6	96.56	RAF1	98.09
PASK	43.15	RET	84.43
PBK/TOPK	101.58	RIPK2	78.07
PDGFRa	49.26	RIPK3	97.51
PDGFRb	40.19	RIPK4	72.63
PDK1/PDPK1	104.84	RIPK5	93.01
PEAK1	57.32	ROCK1	76.28
PHKg1	59.19	ROCK2	88.86
PHKg2	93.88	RON/MST1R	97.92
PIM1	64.95	ROS/ROS1	41.46
PIM2	94.49	RSK1	56.92
PIM3	72.61	RSK2	71.05
PKA	95.33	RSK3	45.64
PKAcb	89.64	RSK4	43.38
PKAcg	86.73	SBK1	101.85
PKCa	76.04	SGK1	95.38
PKCb1	87.40	SGK2	112.94
PKCb2	120.91	SGK3/SGKL	115.42
PKCd	47.64	SIK1	39.74
PKCepsilon	54.70	SIK2	35.75
PKCeta	47.27	SIK3	59.90
PKCg	87.90	SLK/STK2	89.78
PKCiota	88.81	SNARK/NUAK2	45.22
PKCmu/PRKD1	53.10	SNRK	100.39
PKCnu/PRKD3	35.15	SRMS	100.74

Kinase	% Residual Activity	Kinase	% Residual Activity
SRPK1	94.05	ULK2	112.58
SRPK2	95.26	ULK3	82.82
SSTK/TSSK6	79.13	VRK1	42.42
STK16	75.21	VRK2	35.74
STK21/CIT	85.90	WEE1	94.35
STK22D/TSSK1	93.22	WNK1	90.04
STK25/YSK1	97.19	WNK2	94.51
STK32B/YANK2	96.68	WNK3	83.99
STK32C/YANK3	91.06	YES/YES1	70.02
STK33	70.29	YSK4/MAP3K19	63.50
STK38/NDR1	93.33	ZAK/MLTK	83.27
STK38L/NDR2	96.72	ZAP70	100.77
STK39/STLK3	45.71	ZIPK/DAPK3	102.28
SYK	13.43		
TAK1	77.85		
TAOK1	45.50		
TAOK2/TAO1	45.03		
TAOK3/JIK	48.56		
TBK1	94.57		
TEC	100.67		
TESK1	113.17		
TESK2	100.09		
TGFBR2	95.58		
TIE2/TEK	61.19		
TLK1	110.31		
TLK2	97.78		
TNIK	4.38		
TNK1	55.42		
TRKA	14.21		
TRKB	10.81		
TRKC	1.95		
TSSK2	93.78		
TSSK3/STK22C	98.29		
TTBK1	89.46		
TTBK2	92.96		
TXK	60.44		
TYK1/LTK	90.83		
TYK2	93.21		
TYRO3/SKY	82.19		
ULK1	108.01		

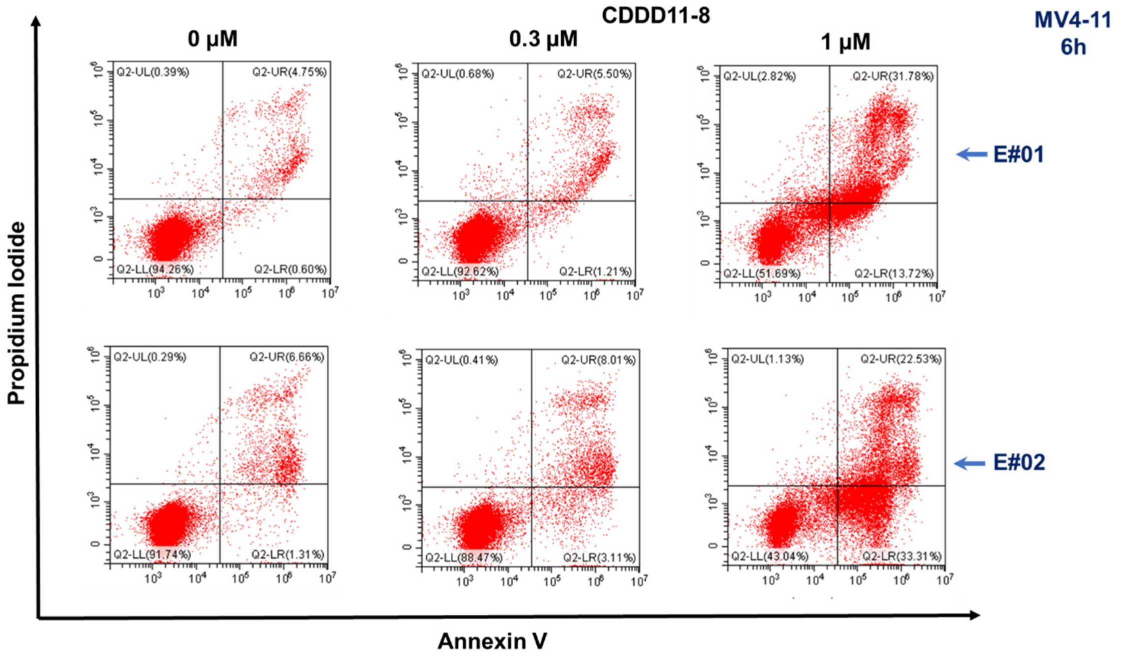

Figure S1: Original flowcytometry data for Figure 2A (MV4-11, 6h, AV)

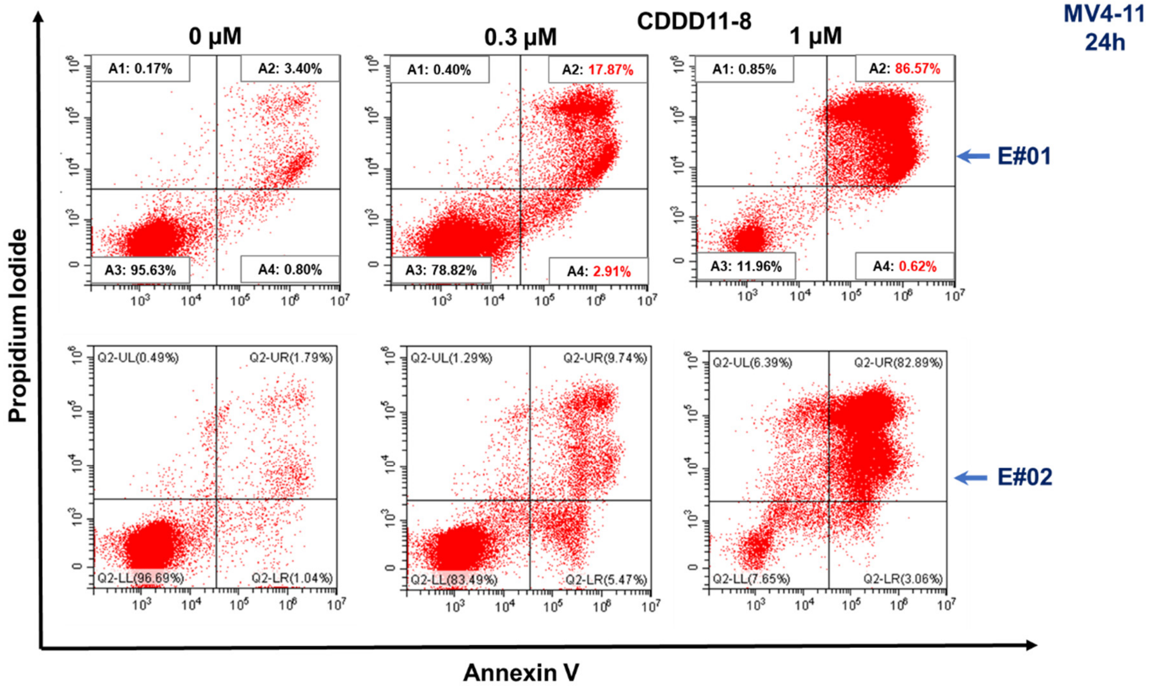

Figure S2: Original flowcytometry data for Figure 2A (MV4-11, 24h, AV)

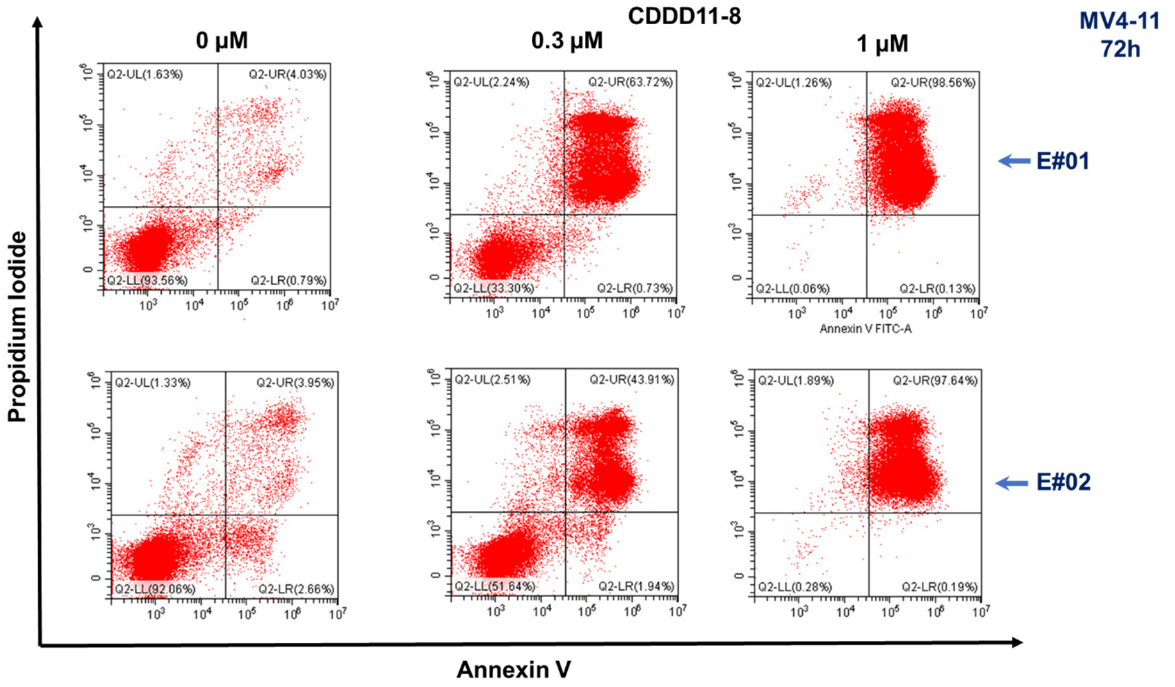

Figure S3: Original flowcytometry data for Figure 2A (MV4-11, 72h, AV)

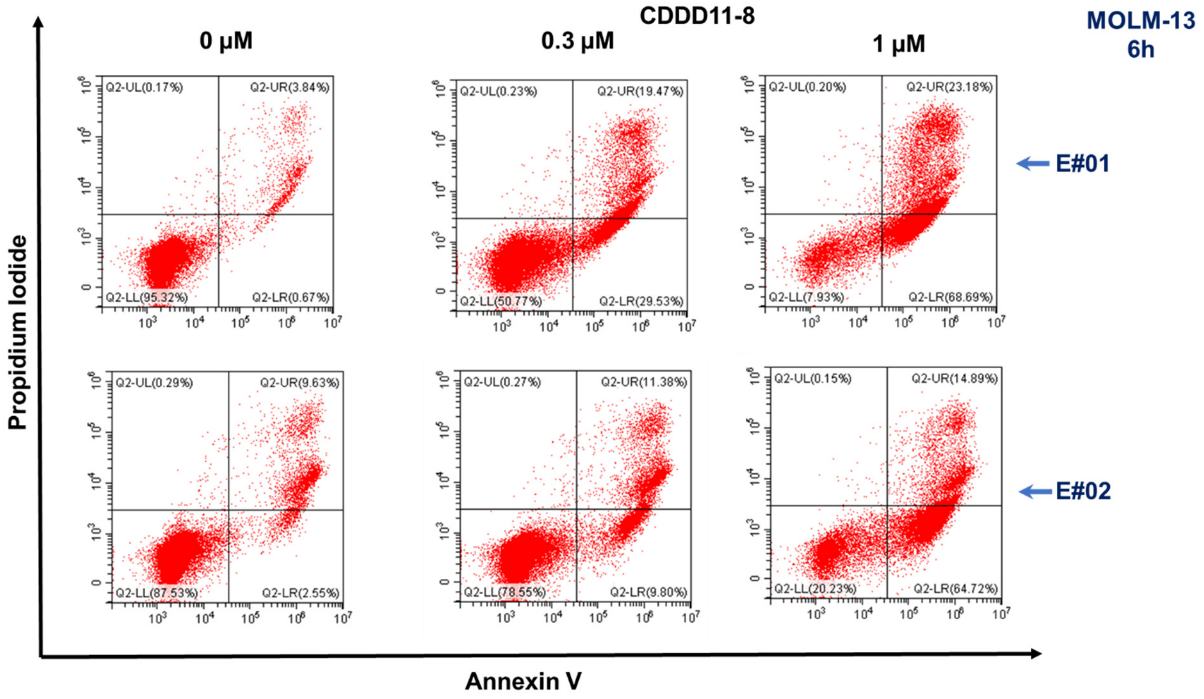

Figure S4: Original flowcytometry data for Figure 2A (MOLM-13, 6h, AV)

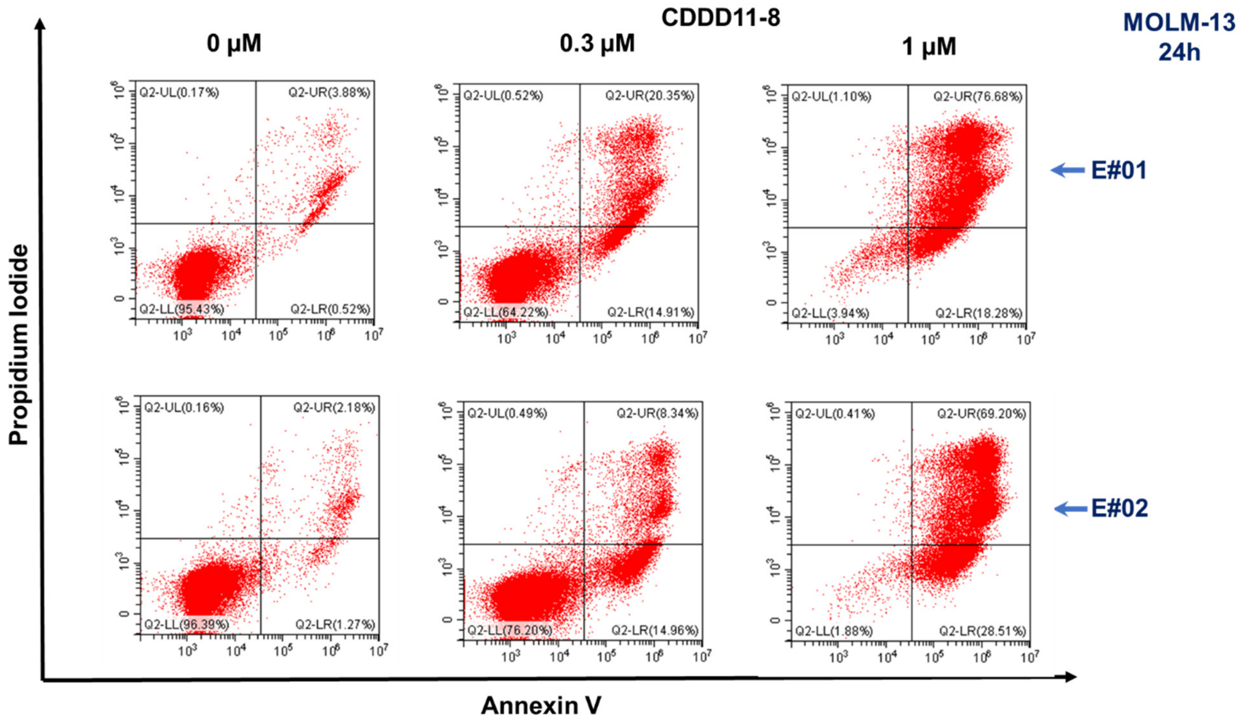

Figure S5: Original flowcytometry data for Figure 2A (MOLM-13, 24h, AV)

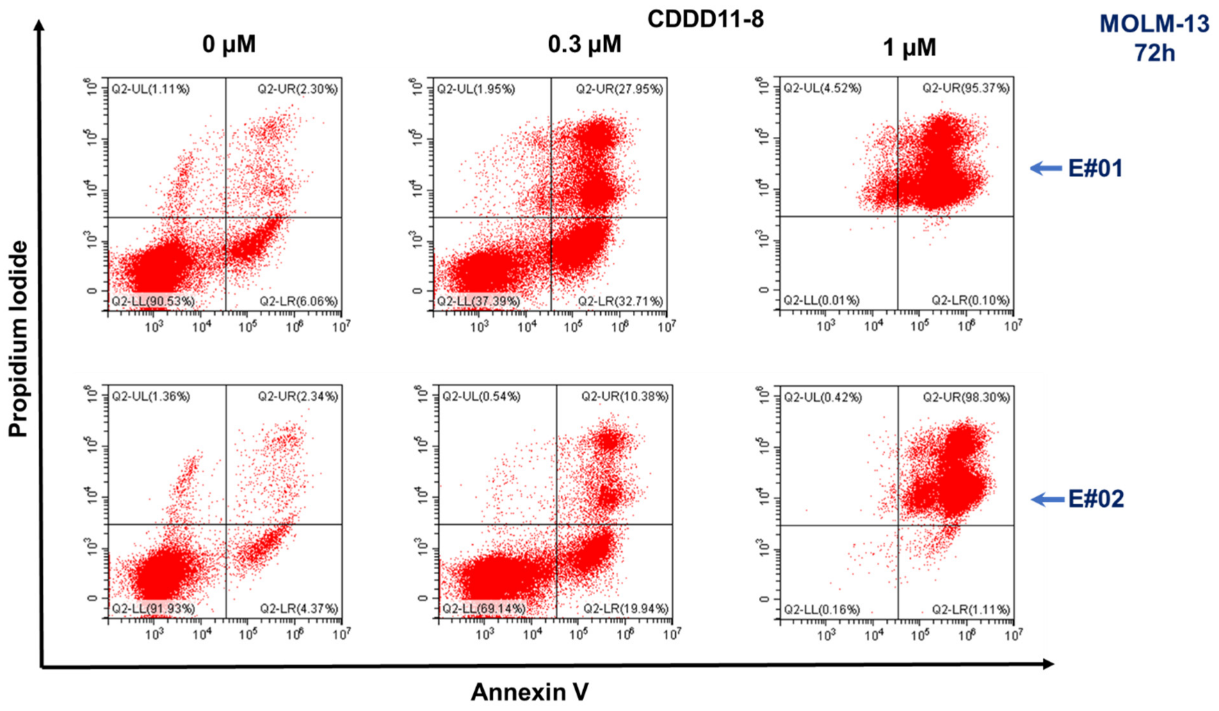

Figure S6: Original flowcytometry data for Figure 2A (MOLM-13, 72h, AV)

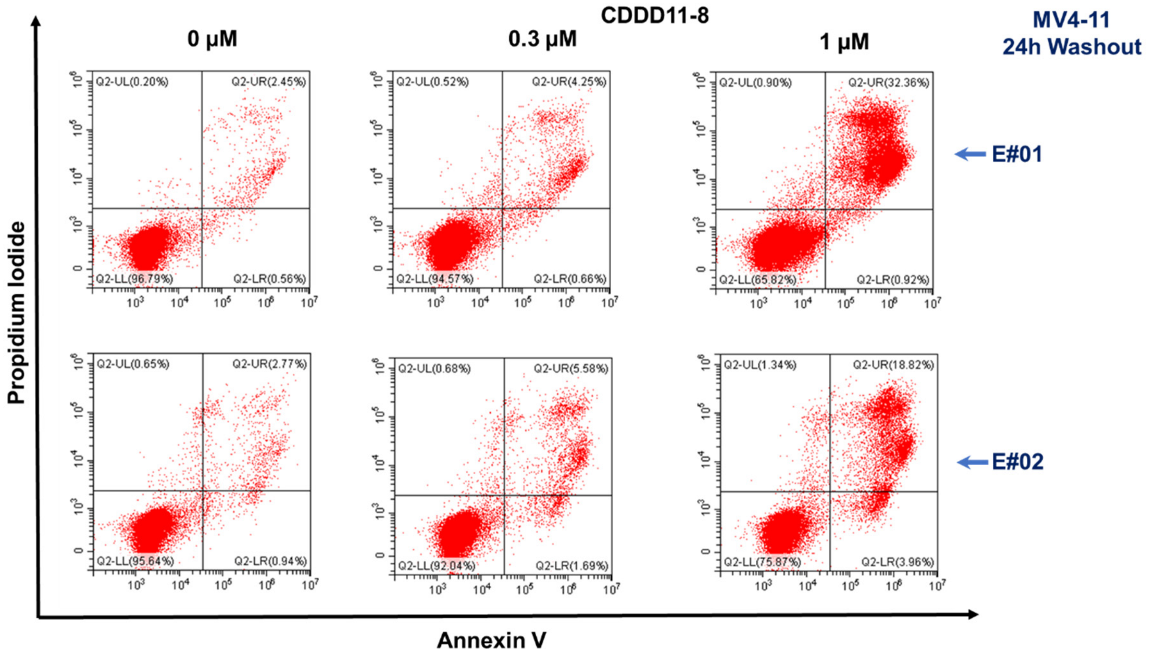

Figure S7: Original flowcytometry data for Figure 2B (MV4-11, 24h washout, AV)

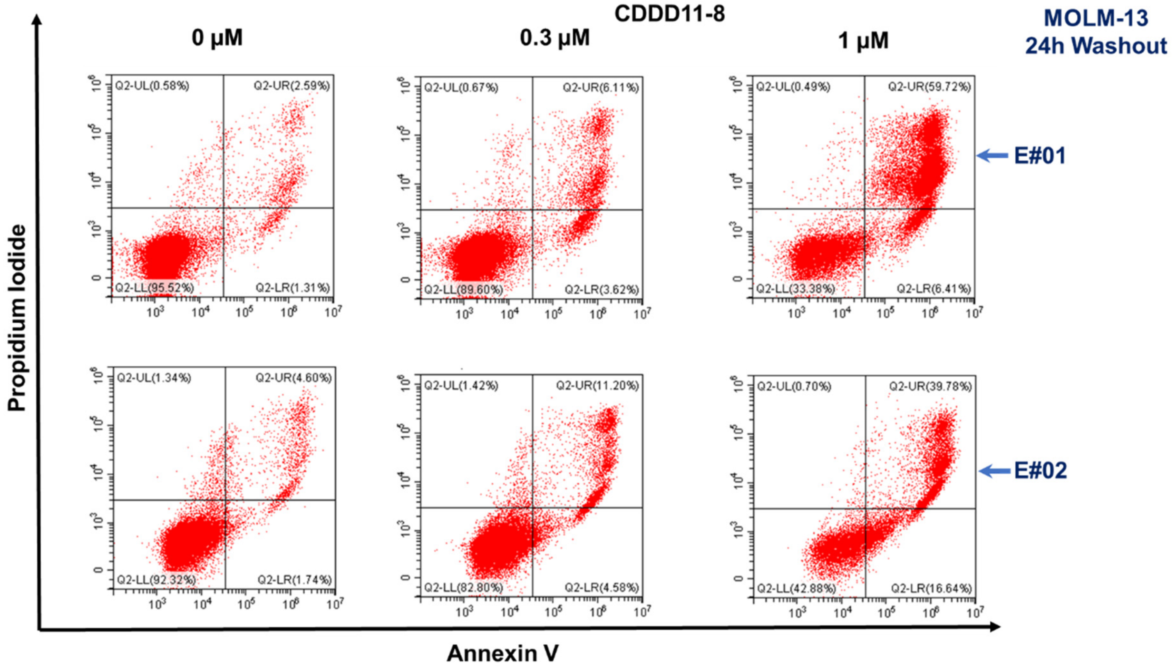

Figure S8: Original flowcytometry data for Figure 2B (MOLM-13, 24h washout, AV)

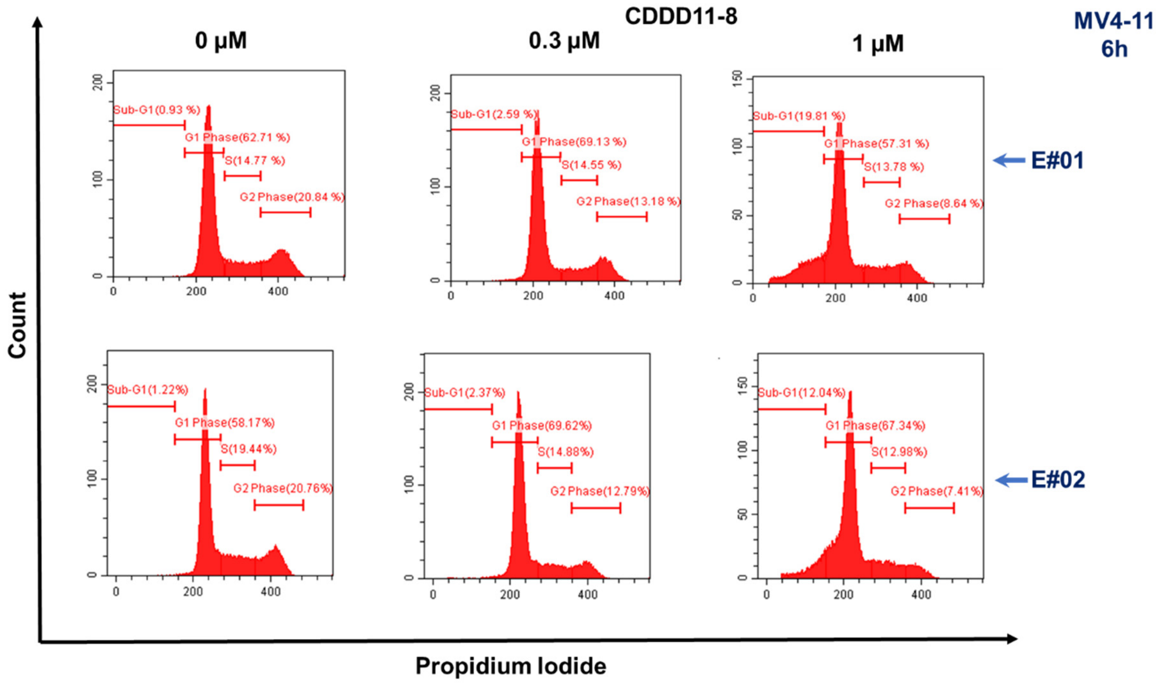

Figure S9: Original flowcytometry data for Figure 2C (MV4-11, 6h, cell cycle)

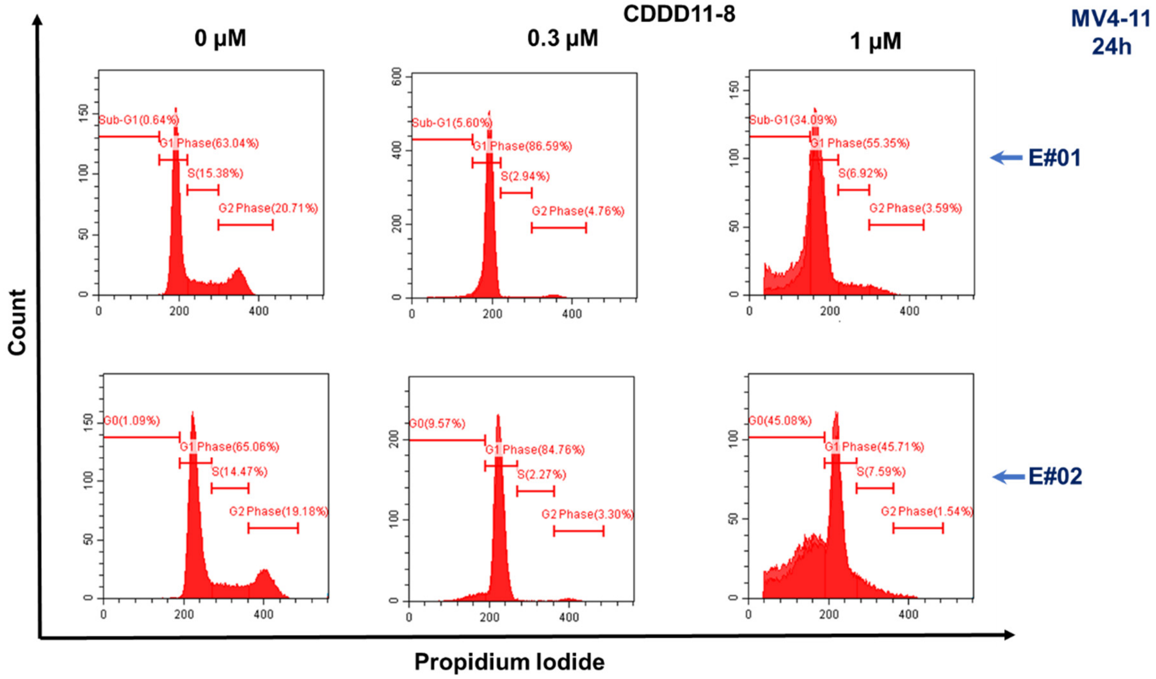

Figure S10: Original flowcytometry data for Figure 2C (MV4-11, 24h, cell cycle)

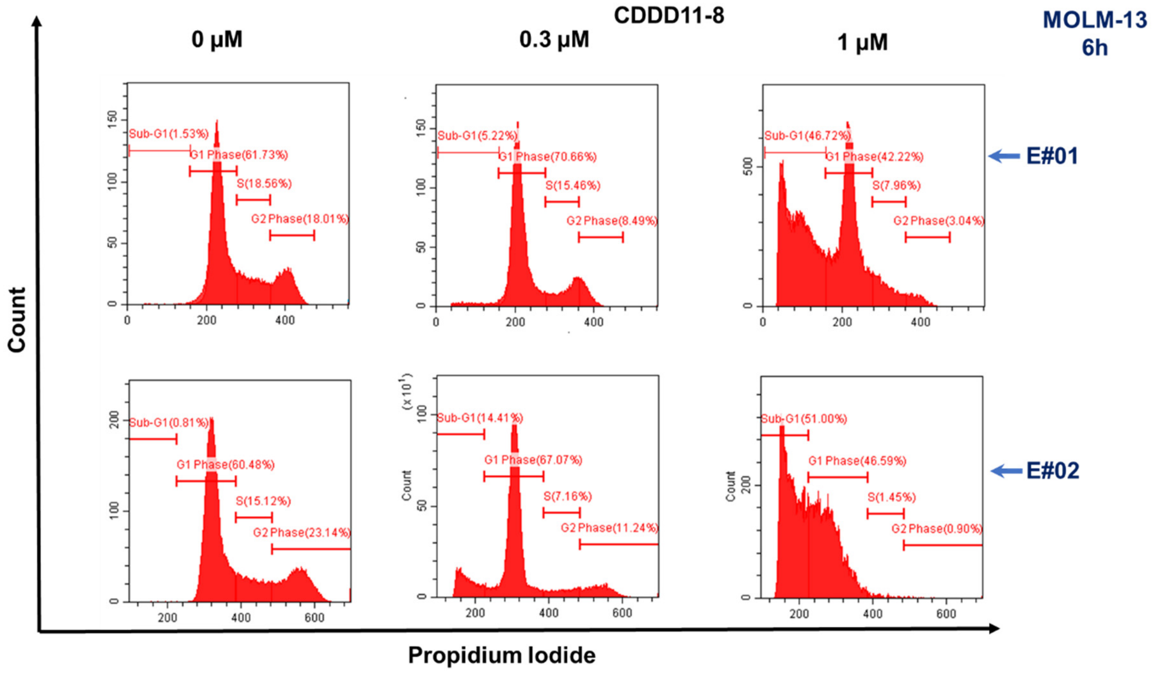

Figure S11: Original flowcytometry data for Figure 2C (MOLM-13, 6h, cell cycle)

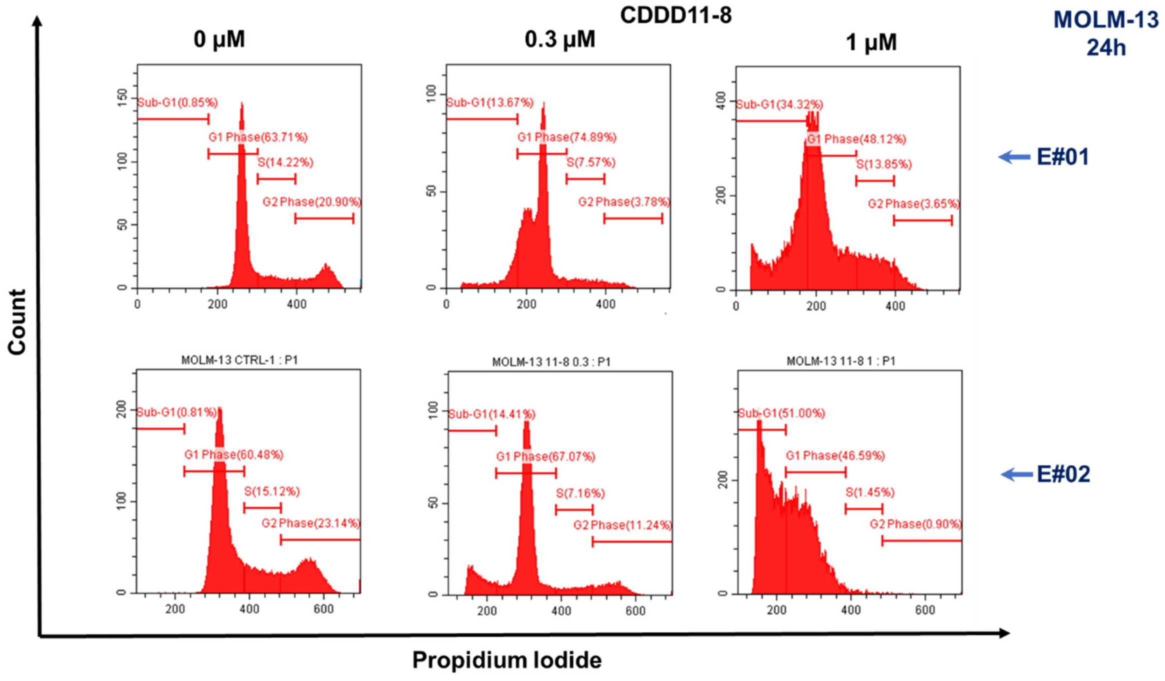

Figure S12: Original flowcytometry data for Figure 2C (MOLM-13, 24h, cell cycle)

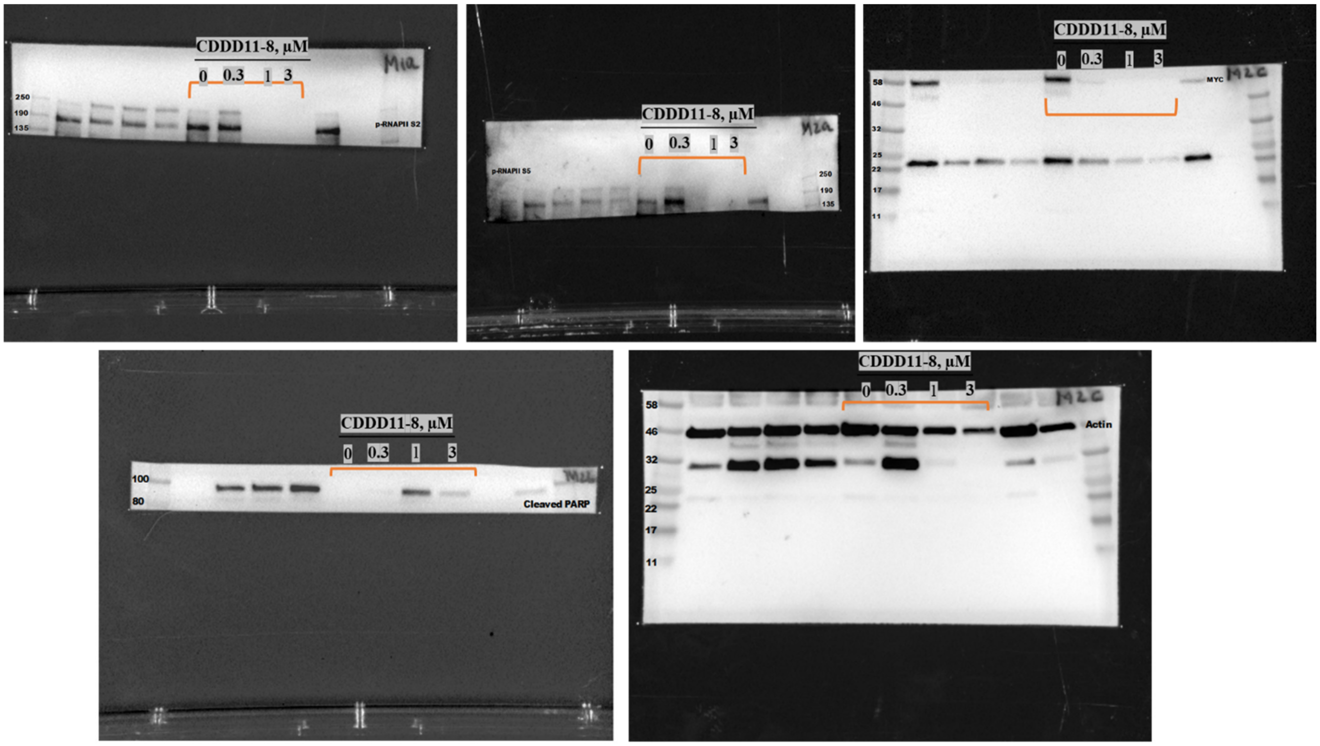

MV4-11, 24h

Figure S13: Uncropped western blots for Figure 3A (MV4-11, 24h, dose-range)

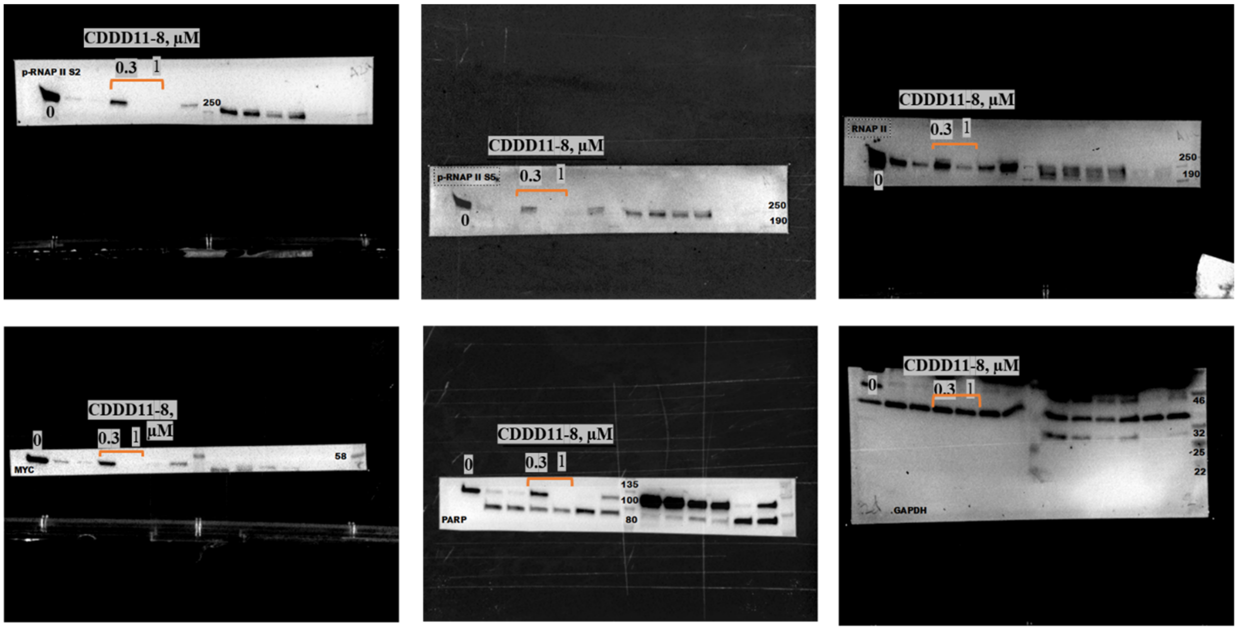

MOLM-13, 24h

Figure S14: Uncropped western blots for Figure 3A (MOLM-13, 24h, dose-range)

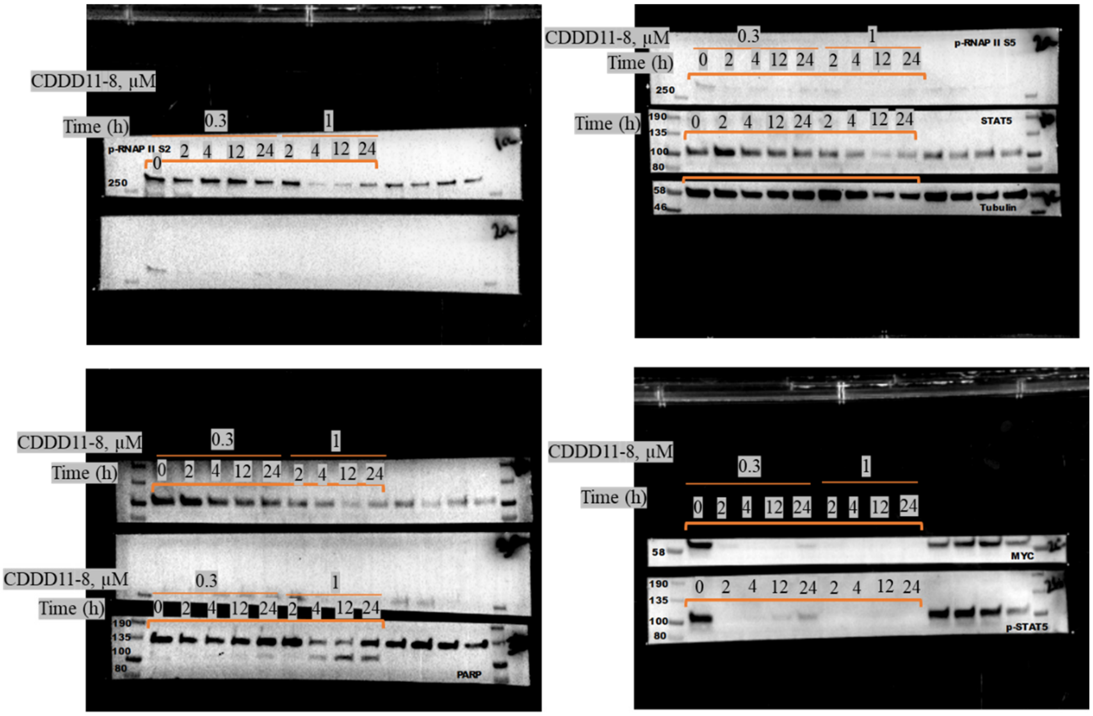

MV4-11 Time course

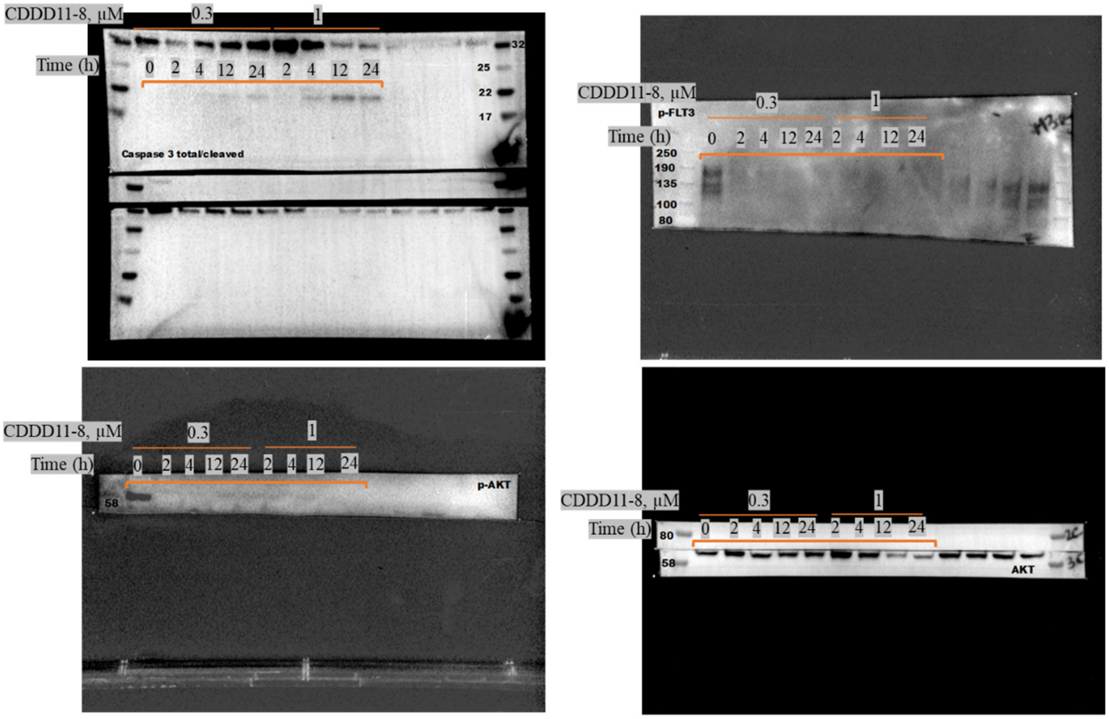

MV4-11 Time course

Figure S15: Uncropped western blots for Figure 3B (MV4-11, time-course)

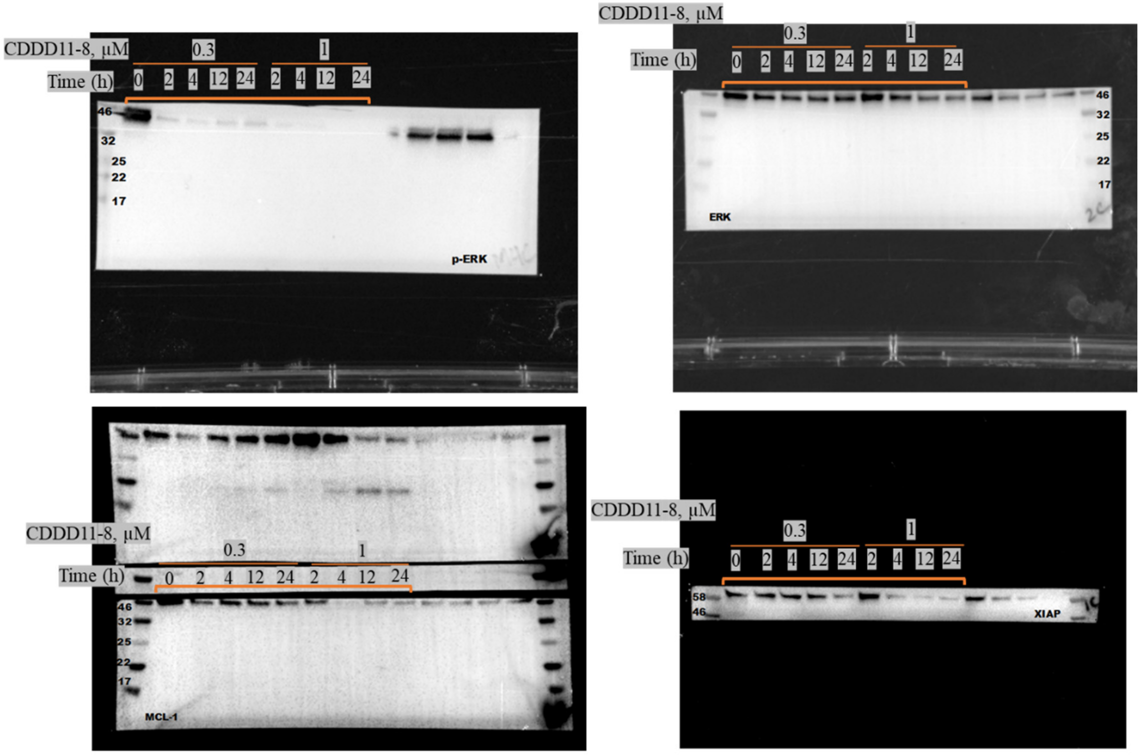

MV4-11 Time course

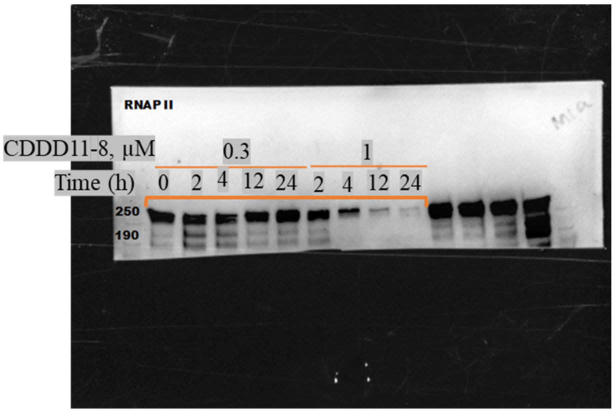

MV4-11 Time course

Figure S15: Uncropped western blots for Figure 3B (MV4-11, time-course)

Table S2: Plasma concentrations of CDDD11-8 in mice after single PO (10 and 100 mg/kg) and IV (2 mg/kg) doses*

PO (10 mg/kg)		PO (100 mg/kg)		IV (2 mg/kg)	
Time (h)	Conc. (ng/mL)	Time (h)	Conc. (ng/mL)	Time (h)	Conc. (ng/mL)
0.13	140	0.10	810	0.03	2288
0.28	300	0.38	925	0.07	2154
0.40	139	0.73	2661	0.10	1313
0.65	389	1.2	1809	0.17	1073
1.2	740	1.9	3007	0.20	1024
1.9	356	2.6	3681	0.30	781
2.6	450	2.9	2485	0.53	367
3.3	95	4.9	4831	0.68	271
4.0	169	6.1	2161	1.4	132
4.7	34	7.0	881	2.1	50
5.4	17	7.8	717	3.4	20
6.2	20	8.3	1201	4.1	11
6.8	3.4	8.9	854	5.4	3
7.8	1.6	9.6	248	6	3.2
8.5	1.8	16.0	77	7	2.4
9.7	1.0	22.1	153	7.7	2.8
15.8	0	24.6	74	8	2.8
21.0	0	29.0	62	8.8	2
24.6	0	41.2	0	9.5	1.7

*Up to three blood samples were collected at non-consecutive times from an individual mouse (right cheek, left cheek, and heart). These individual samples contributed to the overall concentration-time profile of each route. Only non-zero values were included in the final analysis.

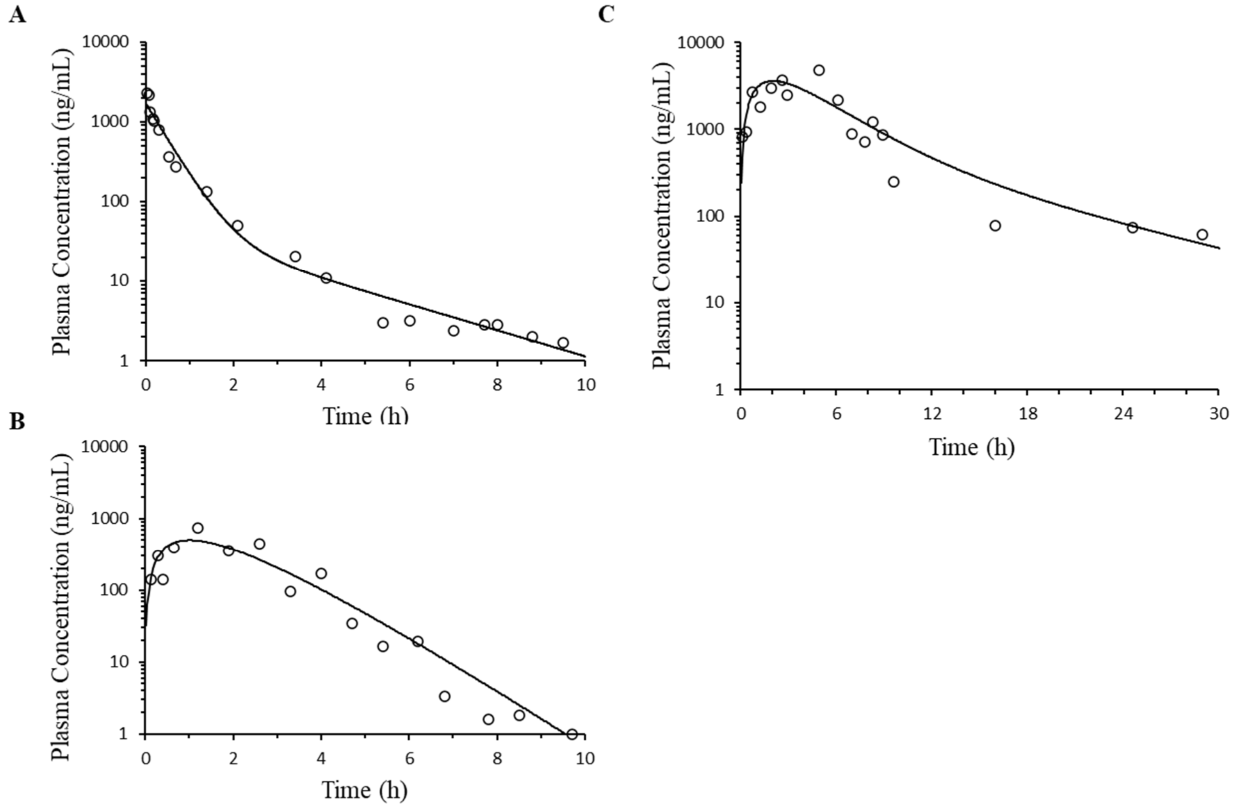

Figure S16: Plasma concentration-time profiles of CDDD11-8 in mice after single **(A)** *IV* dose of 2 mg/kg, PO doses of **(B)** 10 or **(C)** 100 mg/kg.

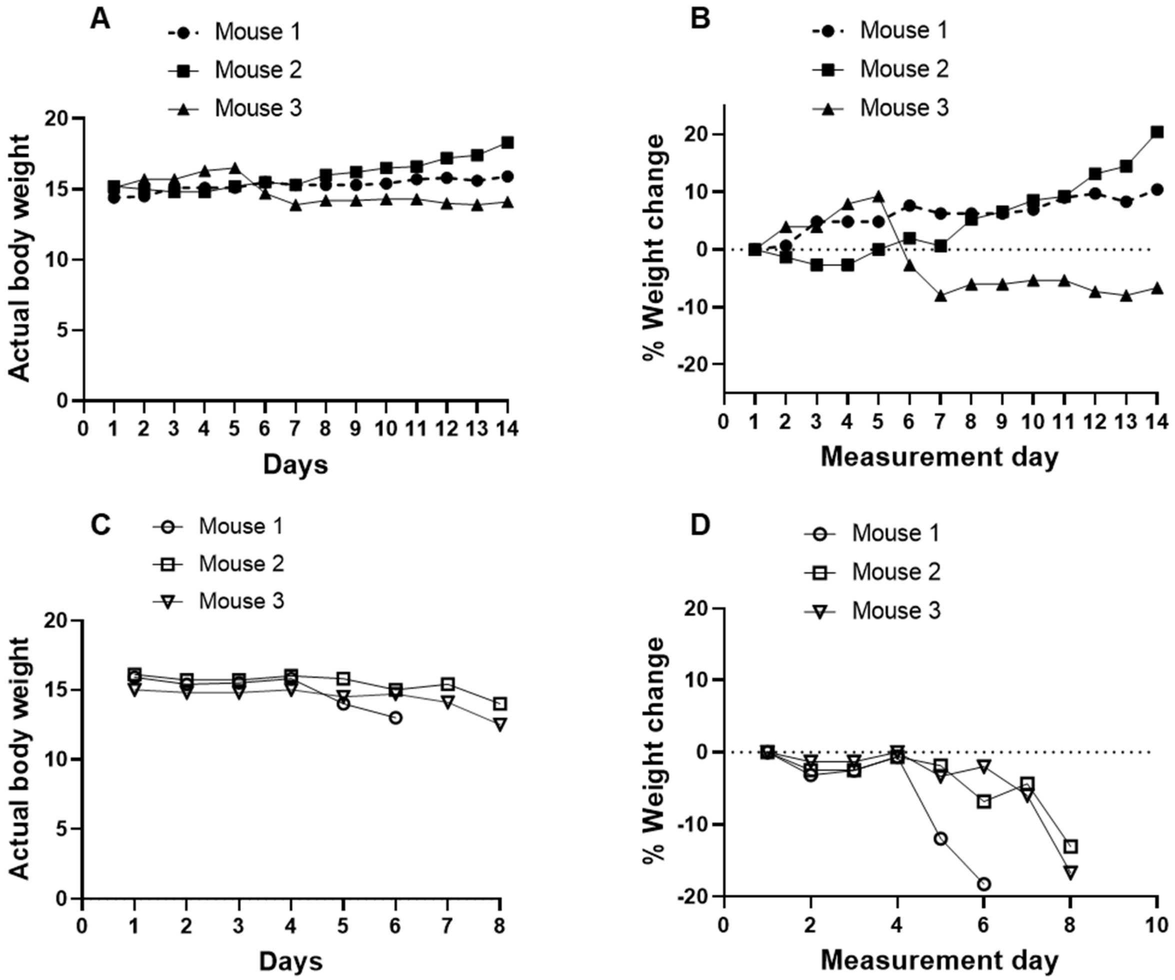

Figure S17: Effect of CDDD11-8 on body weight of mice. Female nude mice (n = 3 per group) were administered oral doses of CDDD11-8 at **(A** and **B)** 150 mg/kg **(C** and **D)** 200 mg/kg per day for 7 days followed by 7 days of follow-up. Body weight was assessed daily. Data presented as mean ± SEM.

Table S3: Tumor volume measurements throughout the treatment period

Measurement days	Individual tumor volume (mm^3)																				
	Vehicle, PO QD							CDDD11-8 75 mg/kg, PO QD							CDDD11-8 125 mg/kg, PO QD						
1	134	101	113	111	130	100	106	117	120	108	129	102	112	116	123	100	120	122	115	124	104
3	146	126	184	150	192	154	146	158	142	93	106	90	128	110	92	87	110	95	111	133	99
5	220	154	212	181	244	147	170	128	130	102	114	86	120	118	90	86	86	96	80	108	84
7	294	175	293	249	285	195	206	138	124	134	141	84	93	138	83	74	86	110	99	107	49
9	372	220	389	261	301	246	177	82	110	83	107	74	48	120	55	55	42	35	26	54	44
11	430	238	391	391	329	301	201	82	93	72	85	50	32	85	57	55	29	20	23	41	37
13	551	271	407	379	377	302	290	44	54	15	74	37	2	93	54	24	0	0	0	1	32
15	573	316	461	403	428	319	295	0	56	7	47	35	0	74	49	24	0	0	0	0	24
17	610	332	541	416	470	360	327	0	66	0	18	25	0	64	37	17	0	0	0	0	2
19	625	368	560	427	554	445	341	0	67	0	40	31	0	43	33	21	0	0	0	0	1
21	846	422	616	508	731	495	412	42	48	0	29	19	0	46	0	30	29	0	0	0	2
23	1137	547	661	622	994	675	473	105	32	0	87	15	0	26	0	32	36	0	0	0	2
25	1237	608	739	797	1188	831	558	186	67	71	124	26	0	64	0	34	25	0	0	0	1
27	1330	761	831	801	1353	986	587	323	79	194	253	30	0	149	2	26	69	0	2	0	0
29	1644	803	943	979	1623	1070	639	418	123	223	315	33	0	239	3	26	125	0	54	0	0

Table S4: Percent tumor growth inhibition throughout the treatment period

	% TGI	
Measurement days	CDDD11-8 75 mg/kg, PO QD	CDDD11-8 125 mg/kg, PO QD
1	-	-
3	92	127
5	101	133
7	95	122
9	115	142
11	121	137
13	127	139
15	129	136
17	128	133
19	125	130
21	119	123
23	112	117
25	105	114
27	96	112
29	92	109

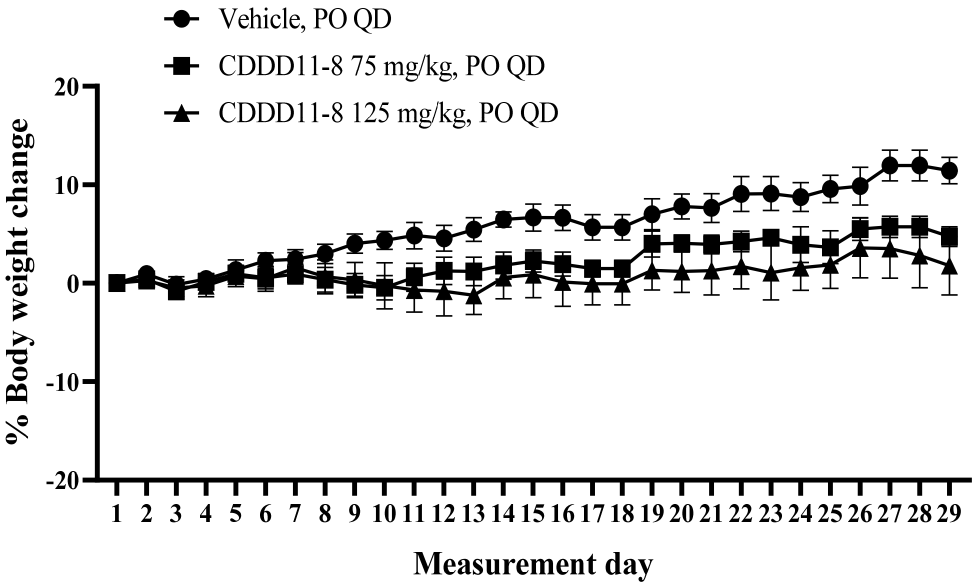

Figure S18: Percent change in body weight of MV4-11 tumor-bearing BALB/c nude (nu/nu) mice treated with once daily doses of vehicle or CDDD11-8 (75 or 125 mg/kg) for 28 days. Body weight was assessed daily. Data presented as mean ± SEM.

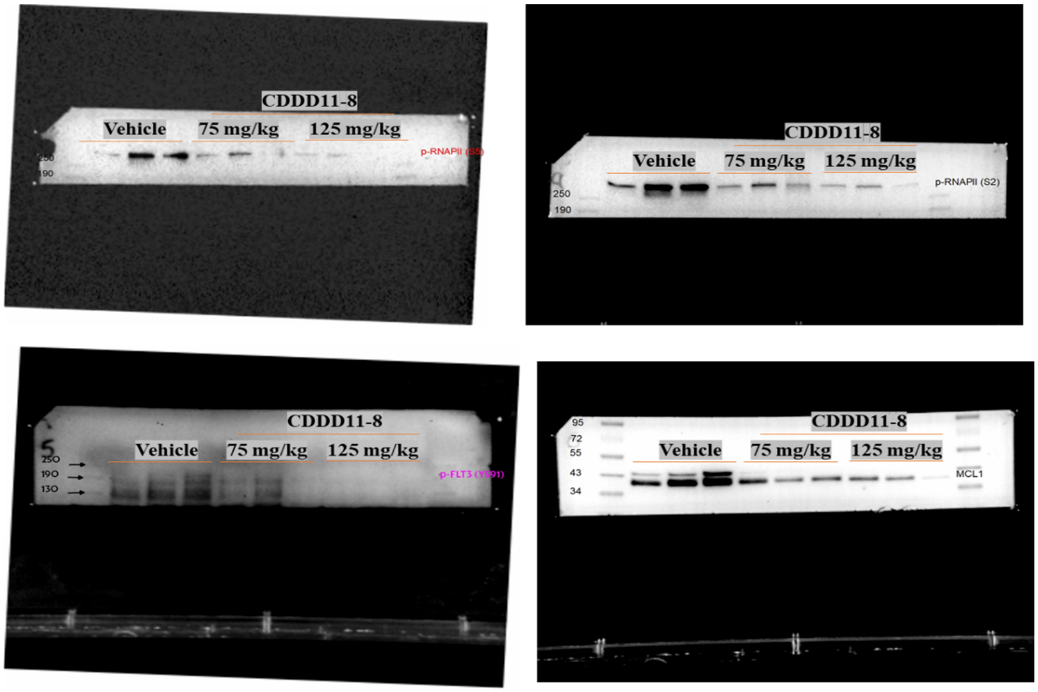

Immunoblotting to study *in vivo* target engagement from MV4-11 tumors

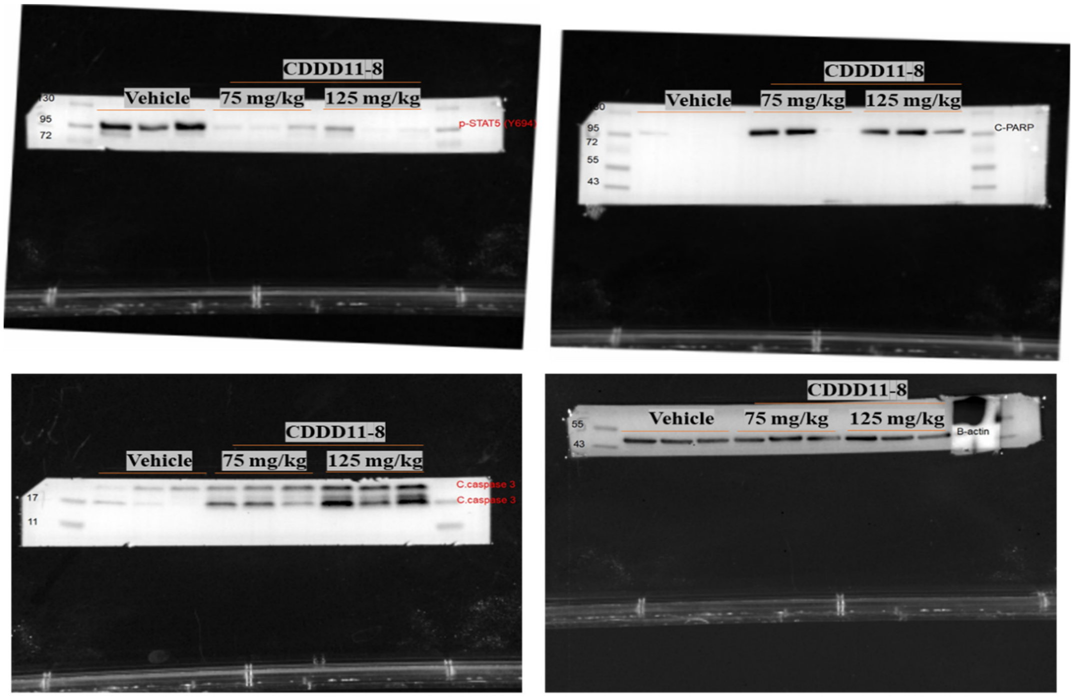

Immunoblotting to study *in vivo* target engagement from MV4-11 tumors

Figure S19: Uncropped western blots for Figure 4C (MV4-11 tumors)

Table S5: Concentration of CDDD11-8 in plasma and tumor 4 h post final dose

Treatment Groups	Mouse ID	Plasma (ng/ml)	Tumor (ng/g)
Vehicle	1	0	0
	2	0	0
	3	0	0
CDDD11-8 75 mg/kg	1	490	2145
	2	441	1776
	3	1091	1481
CDDD11-8 125 mg/kg	1	1438	1676
	2	2849	2312
	3	1795	1851
